# Microbial dynamics along nutrient flow and removal in an integrated multitrophic aquaculture system

**DOI:** 10.3389/fmicb.2026.1781931

**Published:** 2026-04-21

**Authors:** Dzung Nguyen, Ofer Ovadia, Matan Masasa, Andrea Tarnecki, Nathan P. Brennan, Nicole R. Rhody, Kevan L. Main, Lior Guttman

**Affiliations:** 1 Marine Biology and Biotechnology Program, Department of Life Sciences, Ben-Gurion University of the Negev, Eilat, Israel; 2 Department of Life Sciences, Ben-Gurion University of the Negev, Beer-Sheva, Israel; 3 The Goldman Sonnenfeldt School of Sustainability and Climate Change, Ben-Gurion University of the Negev, Beer Sheva, Israel; 4 Morris Kahn Marine Research Station, The Leon H. Charney School of Marine Sciences, University of Haifa, Haifa, Israel; 5 Auburn University Shellfish Lab, Auburn University, Dauphin Island, AL, United States; 6 Marine and Freshwater Aquaculture Research, Mote Marine Laboratory, Sarasota, FL, United States; 7 The Department of Blue Technologies and Sustainable Mariculture, Leon H. Charney School of Marine Sciences, University of Haifa, Haifa, Israel

**Keywords:** microbial dynamics, periphyton, *Ulva*, biofilter, environment heterogeneity, integrated multi-trophic aquaculture (IMTA)

## Abstract

Microbial community assembly in marine integrated multi-trophic aquaculture (IMTA) systems remains poorly understood, particularly across interconnected extractive compartments spanning spatial and temporal scales. Two-step biofilters that incorporate seaweeds and multi-species biofilms (periphyton) are widely used to remove excess nitrogen and phosphorus from aquaculture effluents while simultaneously generating protein-rich, edible biomass. Variations in nutrient composition along these biofilters suggest that microbial diversity and functionality may be differentially shaped within the system. To address this knowledge gap, in this study, using the 16S rRNA gene amplicon sequencing technique, we examined the assembly and potential functions of aquatic microbial communities along the treatment of marine effluent by *Ulva fasciata* and periphyton, where species selection may occur spatially through microbial colonization of the different plant substrates or through changes in water-nutrient content. At the same time, we assessed temporal dynamics by the weekly changes over 5 weeks. Community structure and functionality demonstrated that environmental heterogeneity primarily determined dissimilarity among microbial communities across the biofilter’s compartments. Microbial beta diversity of periphyton, *Ulva* thallus, and rearing water was distinct over time. This confirmed the important role of environmental selection despite hydraulic homogeneity caused by the high dispersal rate of running water within the interconnected biofilters. The periphyton microbial community harbored the highest alpha diversity, followed by the water microbiome and *Ulva*-associated microbiota. In terms of functional potential, nitrogen and sulfur metabolism were generally higher in periphyton than in the water and *Ulva* assemblies. While nitrate reduction by periphyton is associated with the high prevalence of genes involved in denitrification, the *Ulva*-microbes interaction benefits the alga through bacterial dissimilatory nitrate reduction to ammonia. Overall, these findings provide novel insights into the spatial and temporal dynamics of microbiomes in integrated culture systems, contribute to optimal IMTA designs and microbial management in holistic mariculture.

## 1 Introduction

Integrated multi-trophic aquaculture (IMTA) is an ancient, sustainable approach that integrates the cultivation of multiple species, with the waste of one species serving as food for another. Built on this concept, biofilters utilize extractive species like macroalga *Ulva fasciata* and marine periphyton for removing the excess nitrogen and phosphate in fishponds’ effluent (Shpigel et al., 2018; Levy et al., 2017; Shahar and Guttman, 2021). The primary component for nutrient assimilation in such a two-step biofilter is the macroalgae *Ulva,* whose growth, reproduction, and functions are closely dependent on its microbiota (Ghaderiardakani et al., 2017; Nguyen et al., 2023a; Wichard, 2023). The second polishing compartment is marine periphyton, a multi-species biofilm comprising various kingdoms (eukaryotes, prokaryotes, archaea, and viruses) that colonize submerged surfaces (Levy et al., 2017; Nguyen et al., 2023b; Sanli et al., 2015). While the former specializes in ammonia removal, the latter efficiently removes various N forms, including ammonia and nitrate (Guttman et al., 2018). Nutrient availability influences the performance of cultured organisms and may also facilitate changes in their associated microbial communities. While the dynamics of nutrient removal by these two extractive species in IMTA have been extensively examined, our understanding of microbial communities within different compartments of the biofilter, particularly their structure, temporal changes, and functions, is somewhat limited. What is more, many studies emphasized the role of environmental variation (both physical and chemical variables such as T, pH, salinity, DO, nitrate, phosphate) in governing the bacterial community found in various aquatic ecosystems (Yoon et al., 2025; Vitanza et al., 2025; He et al., 2025). Concerning the current research, we hypothesize that nutrients flowing between macroalgae and periphyton might enhance environmental heterogeneity, both due to nutrient gradients among compartments (e.g., ammonia depletion by *Ulva* may promote the presence of different types of bacteria based on their nutrient assimilation capacity) and to microenvironmental differences within each substrate type, thereby creating distinct niches and microbial assemblies. The results will extend our understanding of microbial assembly in a multi-step biofilter, contribute to microbial ecology in aquatic systems in general, and inform better management of mariculture settings in particular.

Research has shown that mariculture generally influences the diversity and metabolism of the associated marine microbes. In IMTA, changes in community composition and structure were evident in both water bodies and sediments, with a predominance of Proteobacteria and Bacteroidetes (Zhang et al., 2024). Concerning *Ulva*, the microbiota associated with this seaweed in an IMTA system has been shown to differ from that found on the wild algae in its natural environment (Califano et al., 2020). Recent studies have also revealed temporal changes in the bacterial community associated with *Ulva* as a biofilter in IMTA (Nguyen et al., 2023a; de Jager et al., 2024), but lack data on the linkage with the water microbiome. This was also the case in previous studies on a marine periphyton biofilter (Nguyen et al., 2023b; Bell et al., 2023). Although studies characterizing microbial communities in aquaculture systems are abundant, few have provided a comprehensive understanding of community dynamics over time, their differentiation across different biofiltration compartments, and their functionality. Still, many of them focus on bacterial-based biofilters, such as those for nitrification or denitrification (Bartelme et al., 2017; Preena et al., 2021). Moreover, although environmental heterogeneity is widely recognized as a crucial factor shaping community assembly in many systems (Langenheder and Lindström, 2019; Nemergut et al., 2013), this conclusion remains unverified in the context of IMTA, where all compartments are interconnected by water fluxes. In the context of the dual biofilter with *Ulva* and periphyton, whether hydraulic connectivity between biofilter compartments promotes dispersal and overwhelms environmental selection, or local environmental conditions in each compartment (i.e., macroalgae vs. periphyton) are strong enough to overcome homogenization despite water exchange, remains a critical question.

Addressing this knowledge gap, the current research examines the compositional and functional variations among microbial assemblies across three ecological niches in the IMTA of water, *Ulva,* and periphyton. The results of this research will contribute to a more comprehensive understanding of microbial assembly in a controlled aquatic environment, thereby enhancing a more holistic approach to mariculture. Throughout the text, we interchangeably use different terms such as “microbial communities,” “microbiomes,” and “assemblies.” When referring to “microbial communities,” it often means a set of co-occurring microorganisms in a defined space and time, whereas “microbiome” refers to the microbial community in a given environment, taking into account its functions. Lastly, “assembly” often refers to a process in which the microbial community is structured over time.

## 2 Materials and methods

### 2.1 Experiment design

**Figure 1:**
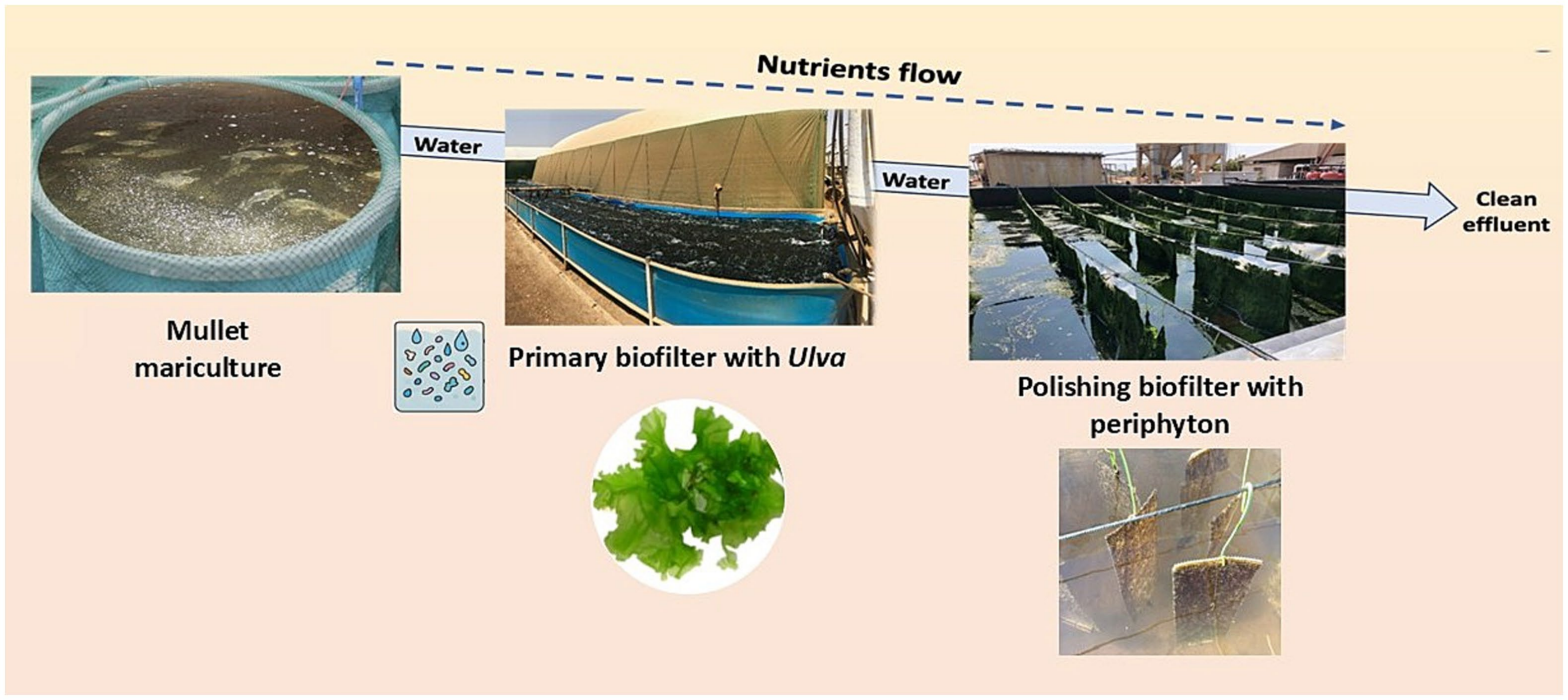
Practical scheme of the dual biofilter system. Effluents from the fish culture were treated by a two-step biofilter, consisting of an upstream compartment containing *Ulva fasciata* and a downstream compartment containing periphyton. Water connects all the compartments, and clean water is led to the sea after biofiltration.

The IMTA with a two-step biofiltration system comprising seaweed (*U. fasciata*) and marine periphyton (Figure 1) was established at the Israel Oceanographic and Limnological Research—The National Center for Mariculture (IOLR-NCM) in the Gulf of Aqaba (Eilat, Israel) according to a previous study (Guttman et al., 2018). Three fishponds (grey mullet *Mugil cephalus*) with a volume of 40m^3^ each were located upstream of the dual biofilter, which received seawater from a location of about 300 meters offshore (32°29′N and 58°34′E) at a depth of 13 meters (Shpigel et al., 2018). The nutrient load of the upstream fishponds remained consistent across all the replicate samples. The two-step biofilter receiving the fishpond effluent consisted of three biological replicates, each with an upstream square tank of 1 m^3^ serving as the *Ulva* biofilter and a similar downstream tank equipped with white plastic nets for the development of marine periphyton (Guttman et al., 2018). The experiment was conducted over five weeks, from March to April 2020. Both the input flow rate and effluent discharge were maintained consistently at approximately 4 m^3^/d. Nutrient concentrations (TAN-N, NO^3^-N, and PO^4^-P) in the water at the inlet and outlet of each biofilter compartment were measured throughout the experiment. Physical parameters (temperature, dissolved oxygen, and pH) were also measured during the experiment (Supplementary Table S1). Such a setting allows effluent from fish tanks to flow through the biofilter, continuously distributing microorganisms to different habitats for settlement, which we will focus on in this study: seaweed (*Ulva*), periphyton, and culture water in each tank.

### 2.2 Samples collection for microbiome analyses

In the aforementioned design, weekly sample collections were performed across different niches, yielding a total of 90 samples for microbiome analyses. Concerning water, samples were collected weekly from the inlets and outlets of the *Ulva* and periphyton biofilters. Samples of 1 L each were collected in sterile bottles and filtered through a 0.47 μm filter under sterile conditions in the laboratory to remove large particles. A subsequent filtration through 0.2 μm filter papers was performed to capture waterborne microbes, and the filters were stored at −80 °C until DNA extraction.

The protocol for sampling the *Ulva*-associated microbiome was as in a previous study (Nguyen et al., 2023a). Shortly, five technical replicates (approximately 20 g each) comprising three thalli of *Ulva* were harvested from each biological replicate on a weekly basis. The samples were transported to the laboratory, where algal thalli were carefully examined to remove any loosely attached sessile organisms. Thalli were then washed in filtered autoclaved natural seawater and stored at −80 °C until DNA extraction.

For periphyton, three plastic nets were randomly collected from each tank on a weekly basis. From each of these nets, a smaller strip 1 cm wide was cut for representation. The collected strips were kept in sterile bags during transportation to the lab in an icebox. Under sterile conditions, biomass was detached from the plastic substrates using published protocols and various tools, including a sterile toothbrush, scraper, and forceps (Nguyen et al., 2023b). Eventually, three samples from each tank were pooled to collect one sample representing one tank, thus making up three biologically replicated samples each week. The collected biomass was finally homogenized, stored in 2 mL tubes, and kept at −80 °C until analysis.

### 2.3 DNA extraction and amplification

For DNA extraction, 200 mg of algal thalli and periphyton were used, utilizing the PureLinkTM Microbiome DNA Purification Kit (Thermo Fisher Scientific) according to the manufacturer’s guidelines. Meanwhile, genomic DNA was isolated from filtered water samples using the DNeasy PowerWater Kit (QIAGEN). The use of different kits was required due to the sample types (water vs. tissue). The bacterial community was characterized using the primer set 515Fa/926R targeting the V4-V5 hypervariable region of the 16S rRNA gene as recommended by the Earth Microbiome Project (Caporaso et al., 2012; Walters et al., 2016). The PCR protocol consisted of an initial denaturation at 95 °C for 5 min, followed by 28 cycles of 94 °C for 45 s, 50 °C for 60 s, and 72 °C for 90 s, and a final elongation at 72 °C for 10 min. PCR products were analyzed via gel electrophoresis in 2.0% agarose, then stored at −20 °C until sequencing.

### 2.4 16S rRNA gene sequencing and processing

Pair-ended sequences of 455 bp length were generated on the Illumina MiSeq platform. FASTQ sequences were uploaded to the NeatSeq-Flow platform, where microbiome analyses were done using QIIME2 (Sklarz et al., 2017; Hall and Beiko, 2018). Quality control was done to discard low-quality sequences (score under 20). OTUs were clustered at a 97% sequence similarity threshold using DADA2 output sequences (Callahan et al., 2016). The taxonomy of these OTUs was then assigned using the SILVA 138.1 database (Quast et al., 2012). Chloroplasts and mitochondria were filtered from the data before analysis. The functional potential prediction was made using PICRUSt based on Greengenes IDs (Douglas et al., 2018). The resulting OTUs and gene abundance tables were then further analyzed using the web-based tool MicrobiomeAnalyst to calculate ecological indices, including alpha and beta diversities (Chong et al., 2020). Pathways related to nitrogen metabolism and biofilm functions were identified using the KEGG Orthology database (Aoki-Kinoshita and Kanehisa, 2007).

### 2.5 Data analysis

Before analyzing the microbiome data, samples were rarefied to a minimum library size of 1,375 OTUs, based on species richness (Supplementary Figure S1). The principal component analysis (PCA) biplot was constructed by using the OTU abundance table and the tables of chemical and physical parameters. Alpha diversity, based on the Shannon Index, was calculated for samples grouped by source (water, *Ulva*, periphyton). A Kruskal-Wallis test was performed to examine differences in alpha and functional diversity across the three habitats and the five time points. The communities’ Beta diversity was determined using Permutational Multivariate Analysis of Variance (PERMANOVA) based on Bray-Curtis metrics, and the results were then subjected to ordination using principal coordinates analysis (PCoA). Multiple comparisons between different KEGG pathways were done using a two-way ANOVA followed by Tukey’s post-hoc test. All statistical tests were performed in R version 4.2.0 using the vegan package version 2.6–2 (Oksanen et al., 2022).

## 3 Results

### 3.1 Environmental heterogeneity as a factor underlying microbial assembly

**Figure 2:**
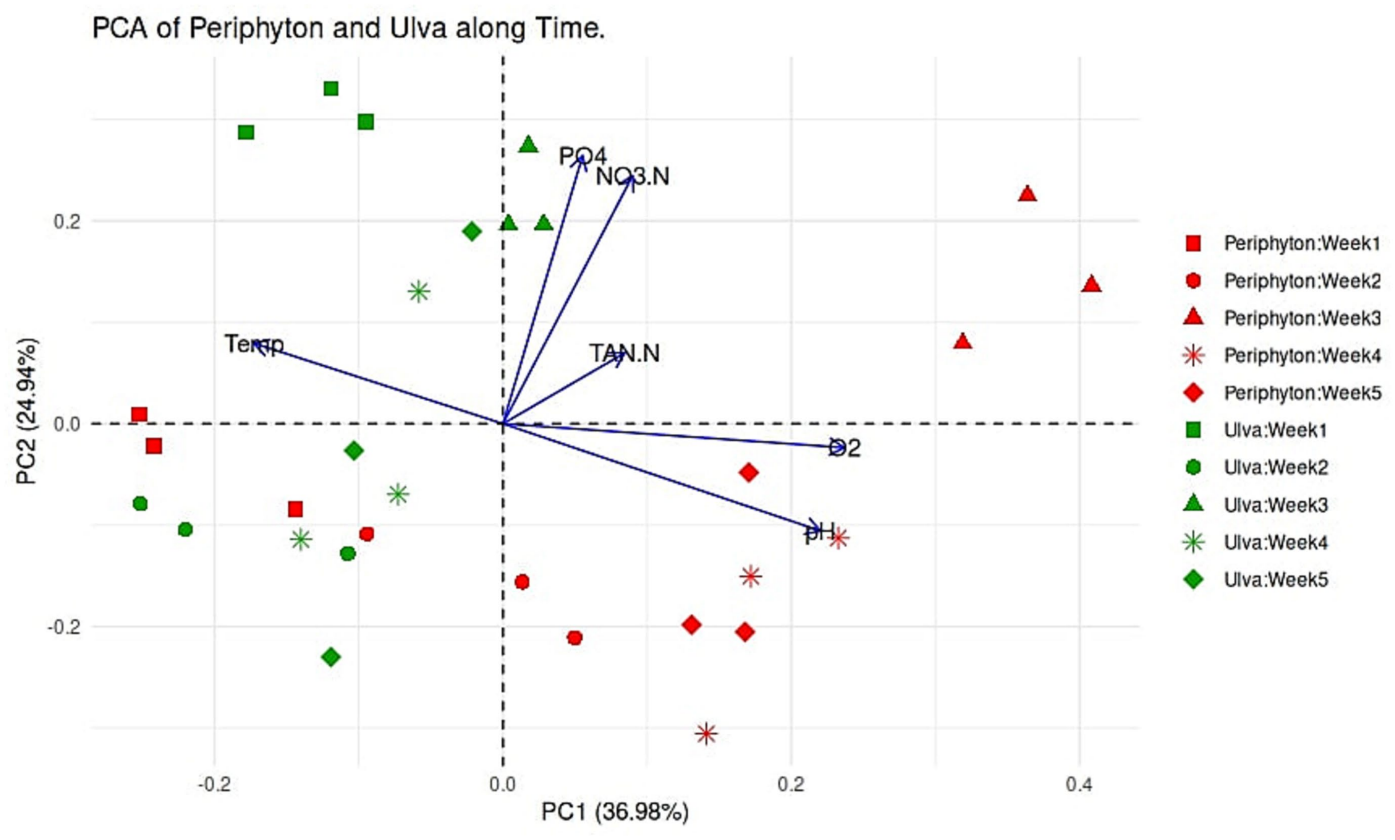
Biplot of the principal component analysis (PCA) showing the environmental parameters measured in the ambient water and microbial communities in marine periphyton and macroalgal *Ulva*. Samplings were made during a five-week experiment. The graph’s green color represents the *Ulva* microbiota, while the red represents the periphyton microbial community. Different shapes indicate different sampling times (weeks 1–5).

The PCA biplot revealed a partitioning between *Ulva* microbiota and periphyton samples at different developmental stages (Figure 2). With respect to environmental chemistry, there were overall positive correlations between pH and oxygen, and between nitrate and phosphate, whereas temperature showed the opposite pattern. These environmental factors significantly contributed to clustering microbiomes from the different cultures of *Ulva* and periphyton. pH and oxygen levels were crucial in explaining the separation of periphyton from *Ulva,* particularly during the late developmental phase (weeks 4 and 5), when periphyton microbiomes also differed from those of the earlier developmental stage (weeks 1 to 3). Nitrate and phosphate were fundamental for the distinguishing of *Ulva*-associated microbial communities during weeks 1 to 3 from other *Ulva* microbiomes in the later culture period and from periphyton microbiomes. In contrast to the aforementioned, TAN and temperature showed no notable correlations with any other water quality parameters or with any specific microbiome of *Ulva* or periphyton. The PCA plot demonstrated separation of marine periphyton by maturation stage, with distinct clusters for the early developmental stage during the first 2 weeks and for the maturation stage at weeks 4 and 5. In the case of the *Ulva* microbiome, microbiomes clustered during the first 3 weeks of culture but then appeared scattered in the weeks after (4 and 5). Generally, the PCA plot of physical and chemical parameters indicated environmental and temporal variations among samples.

### 3.2 Compositional diversity of microbial communities associated with the biofilters

**Figure 3:**
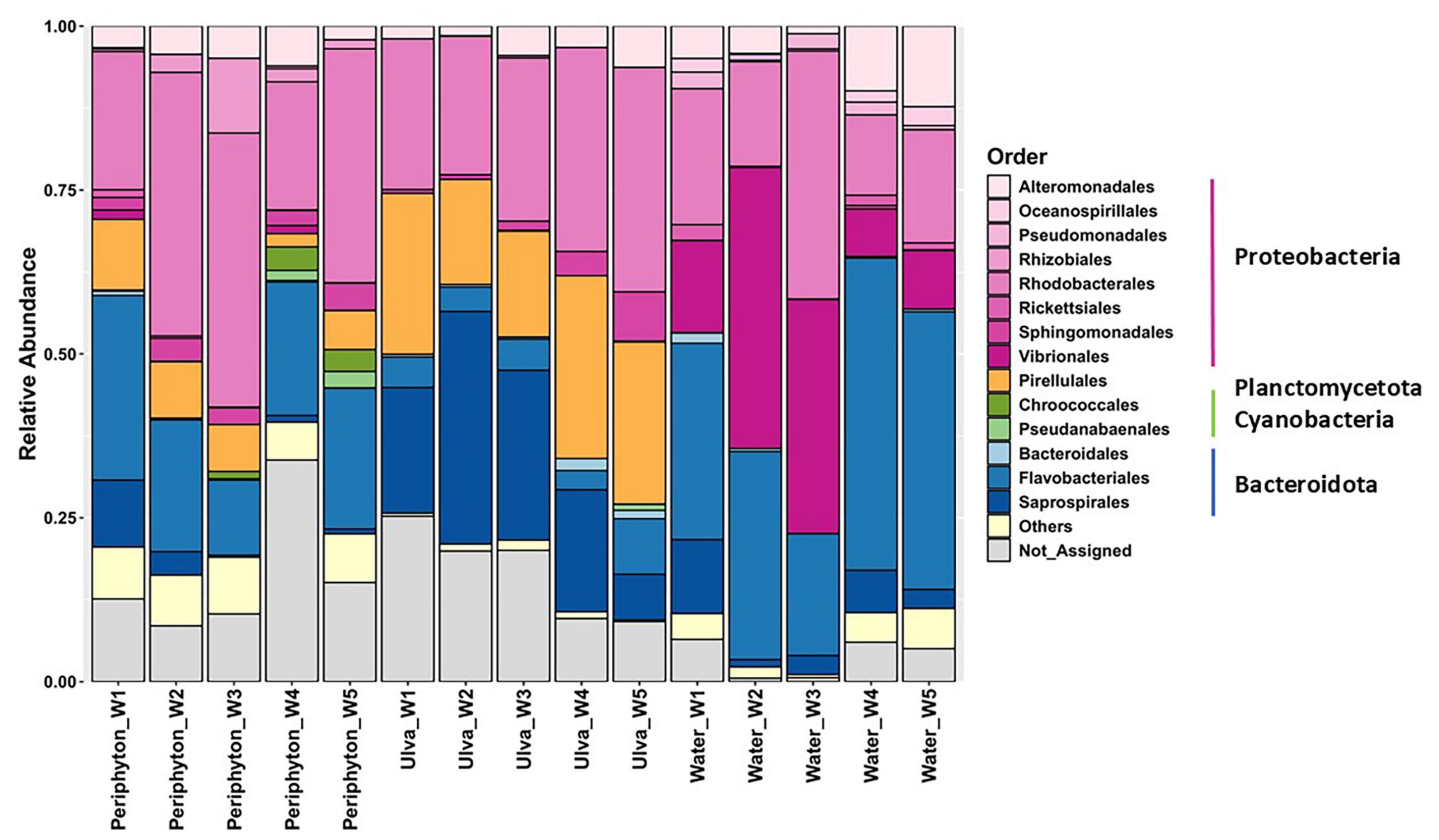
Relative abundance of the microbial community associated with *Ulva*, water, and periphyton during five weeks of succession, shown by the relative abundance at the order level and phylum levels. Each column indicates average data from all the samples of each week.

A total of 1,373 OTUs were found in all three studied habitats. The taxonomic profile revealed distinct microbial community structures across environmental types. At the phylum level, four phyla, Proteobacteria (53.61%), Bacteroidetes (26.03%), Cyanobacteria (9.18%), and Planctomycetes (7.5%), accounted for 96.32% of the total phyla found in the periphyton microbial community. The dominant taxa, which comprised 95% of the *Ulva*-associated microbiome, were Bacteroidetes (36.26%), Proteobacteria (35.27%), Planctomycetes (22.24%), and Cyanobacteria (1.05%). Proteobacteria (58.58%) and Bacteroidetes (39.7%) constituted up to 98.3% of the water microbiome (Supplementary Figure S2, Supplementary Table S2). At the order level, Rhodobacterales is shared across all three habitats as a predominant taxon, with relative abundances of 31.67, 26.87, and 20.81% in periphyton, *Ulva*-associated, and water microbial communities, respectively. While Flavobacteriales were commonly found in periphyton (20.30%) and water (34.05%), Pirellulales stayed in the top three dominant orders in both periphyton (6.89%) and *Ulva*-microbiome (21.87%). On the other hand, Vibrionales dominated water samples with a relative abundance of up to 21.77% (Figure 3, Supplementary Table S3).

**Figure 4:**
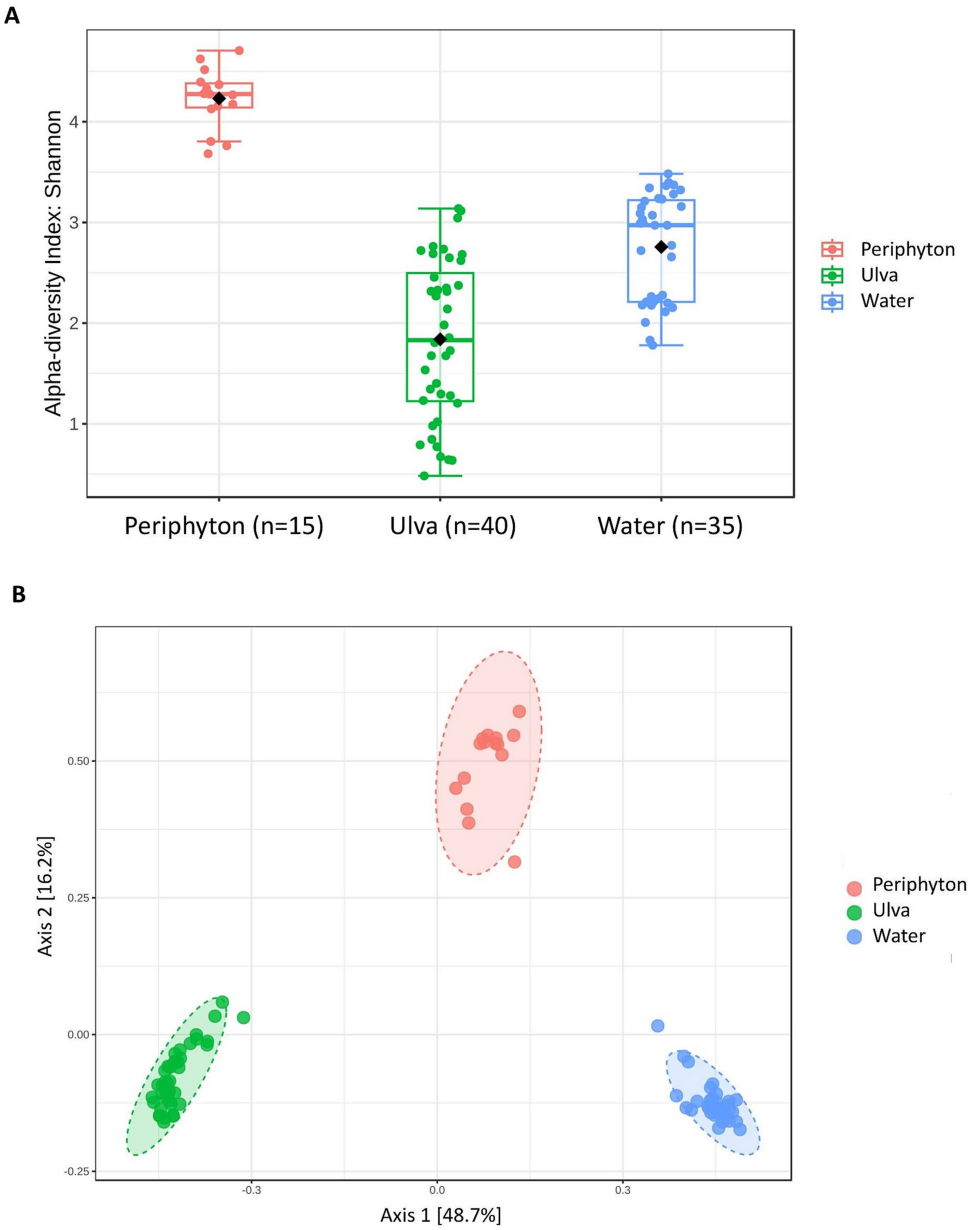
**(A)** Shannon alpha diversity of three microbial communities associated with periphyton, *Ulva*, and water was examined at the feature level; black dots show the average values; Kruskal-Wallis’ statistic (H) value = 52.22, *p* < 0.001 (*n* = 90). **(B)** Dissimilarities in microbial communities across three compartments (periphyton, *Ulva,* and water). Statistical test PERMANOVA based on Bray-Curtis metrics, then subjected to ordination by PCoA; *F*-value = 76.85, *p* = 0.001.

Shannon alpha diversity of the microbial communities in three local habitats, i.e., periphyton, *Ulva*, and water, significantly differed (Figure 4A, Kruskal-Wallis statistical test, *H*-value = 52.22, *p* < 0.001, *n* = 90). The highest diversity was found in periphyton (Shannon diversity index, average H′ = 4.2), followed by water (average H′ = 2.7) and then *Ulva* microbiome (average H′ = 1.8). Likewise, beta diversity based on Bray-Curtis indices revealed distinct clusters among samples from different local habitats (Figure 4B; PERMANOVA, *F*-value = 76.85, *p* = 0.001). Notably, these dissimilarities aligned with the clustering of communities from other compartments when phylogenetic relationships were considered (Weighted UniFrac, Supplementary Figure S3, PERMANOVA, *p* = 0.001).

### 3.3 Differentiation of microbial communities across three hosts through time

**Figure 5:**
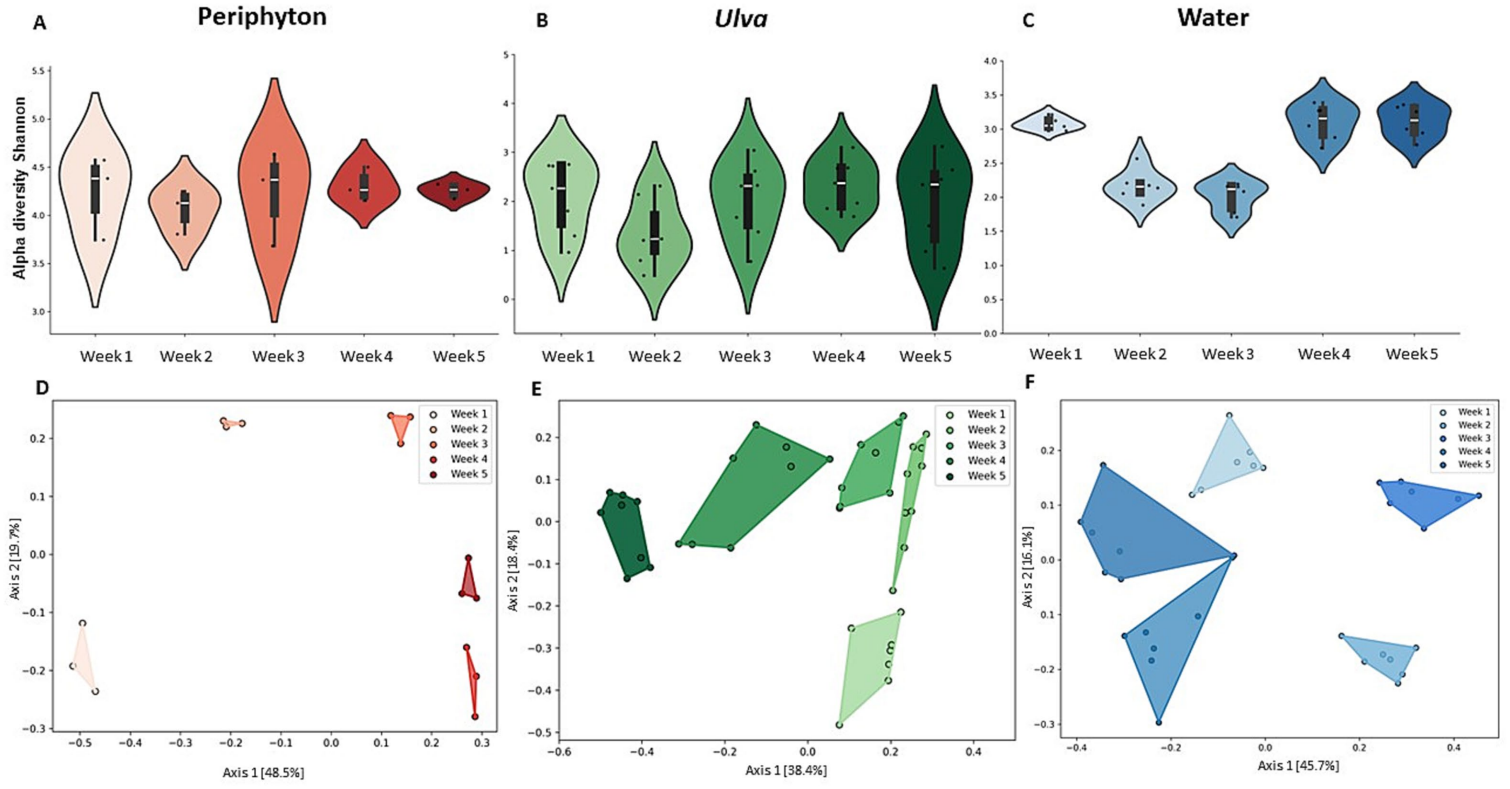
Alpha Shannon diversity of the bacterial communities across three compartments of periphyton **(A)**, *Ulva* **(B)**, and water **(C)** following 5 weeks. Kruskal-Wallis’ test was performed in three cases with respective results of *H* = 9.37, *p* = 0.053 (*n* = 15); *H* = 15.05, *p* < 0.005 (*n* = 40); and *H* = 24.68, *p* < 0.001 (*n* = 35). Box plots show variation between samples of each week; white dashes inside the boxplots are mean values. Dissimilarities based on Bray-Curtis’ indices of the microbial communities associated periphyton **(D)**, *Ulva* **(E)**, and water **(F)**, along a five-week experiment. PERMANOVA statistical test for three habitats resulted in *F*-value = 14.31, *p* = 0.001 (*n* = 15); *F*-value = 14.12, *p* < 0.001 (*n* = 40); and *F*-value = 21.62, *p* = 0.001 (*n* = 35), respectively, then subjected to ordination by PCoA.

When temporal force was considered, all three habitats exhibited fluctuations in Shannon alpha diversity. However, only the *Ulva* and seawater habitats revealed a significant change along temporal dimensions, while this was not identified *Ulva* in the periphyton (Figures 5A–C; Kruskal Wallis’ test, *H*-value = 9.37, *p* = 0.053 (*n* = 15); *H*-value = 15.05, *p* < 0.005 (*n* = 40), and *H*-value = 24.68, *p* < 0.001 (*n* = 35), respectively). In marine periphyton, the highest and lowest values of H′ index, varied between 4.5 and 3.9, in weeks 1 and 4, respectively; while in the *Ulva*-associated microbiome H′ index was reduced by 2.5 folds between weeks 2 and 4. In seawater, alpha diversity of the first and last weeks was high (H′ = 3.2), while the lowest diversity was recorded in the middle of succession (H′ = 2.1). Among all types of types, the *Ulva* microbiome showed the highest variation among biological replicates at each time point (Alpha Shannon diversity, standard deviation of 0.79 in *Ulva* versus 0.53 in water and 0.28 in periphyton). Noticeably, beta diversity based on Bray-Curtis proved that in all three local habitats of periphyton, *Ulva,* and water, the microbial succession was characterized by distinct communities over time (Figures 5D–F; PERMANOVA, *F*-value = 14.31, *p* = 0.001 (*n* = 15); *F*-value = 14.12, *p* < 0.001 (*n* = 40); and *F*-value = 21.62, *p* = 0.001 (*n* = 35), respectively).

### 3.4 Predicted functional diversity in microbial communities harboring the dual biofilters

**Figure 6:**
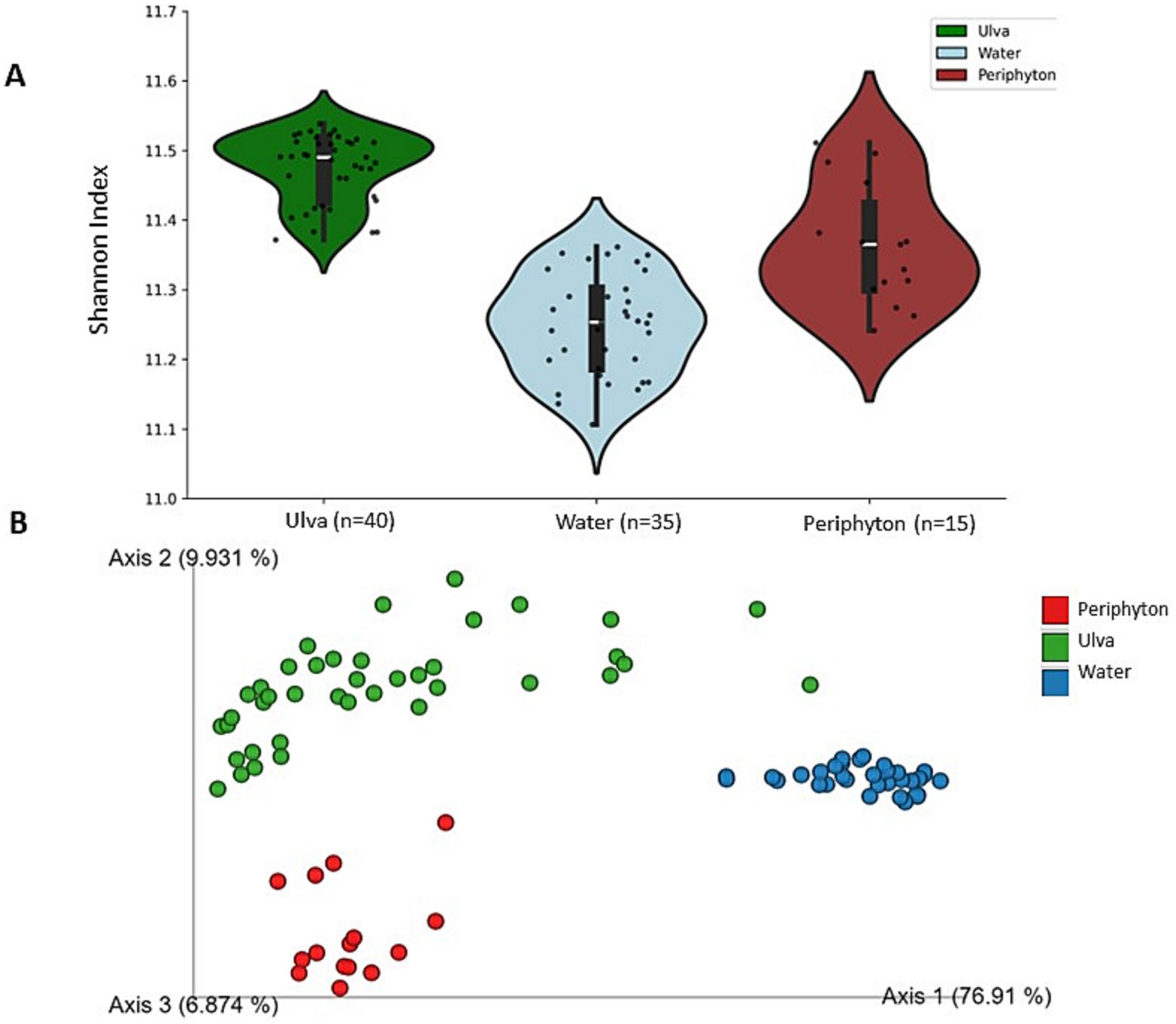
**(A)** *alpha* functional diversity of three microbial communities associated with periphyton, *Ulva*, and water was examined at the feature level; black dots show the average values; Kruskal-Wallis’ statistic (H) value = 63.6, *p* < 0.001 (*n* = 90). **(B)** *beta* functional diversity across three compartments (periphyton, *Ulva*, and water). Statistical test PERMANOVA based on Bray-Curtis metrics, then subjected to ordination by PCoA; *F*-value = 125.74, *p* = 0.001 (*n* = 90).

Likewise taxonomic diversity, a significant difference in the functional diversity was also obtained among the three microbial communities. Shannon’s alpha functional diversity of the *Ulva* microbiome was the highest, followed by periphyton and then the water microbiome [Figure 6A, Kruskal Wallis’ test, *H*-value = 63.6, *p* < 0.001 (*n* = 90)]. Beta functional diversity measured by Bray-Curtis’ indices indicated clear separation among samples representing different habitats [Figure 6B, PERMANOVA, F-value = 125.74, *p* = 0.001 (*n* = 90)]. The dendrogram further supported this result, illustrating that samples from various origins were grouped into three clades (Supplementary Figure S4A). Additionally, a clustered heatmap of predicted functions annotated by the KEGG database highlighted the different gene abundance found in the three communities (Supplementary Figure S4B).

**Figure 7:**
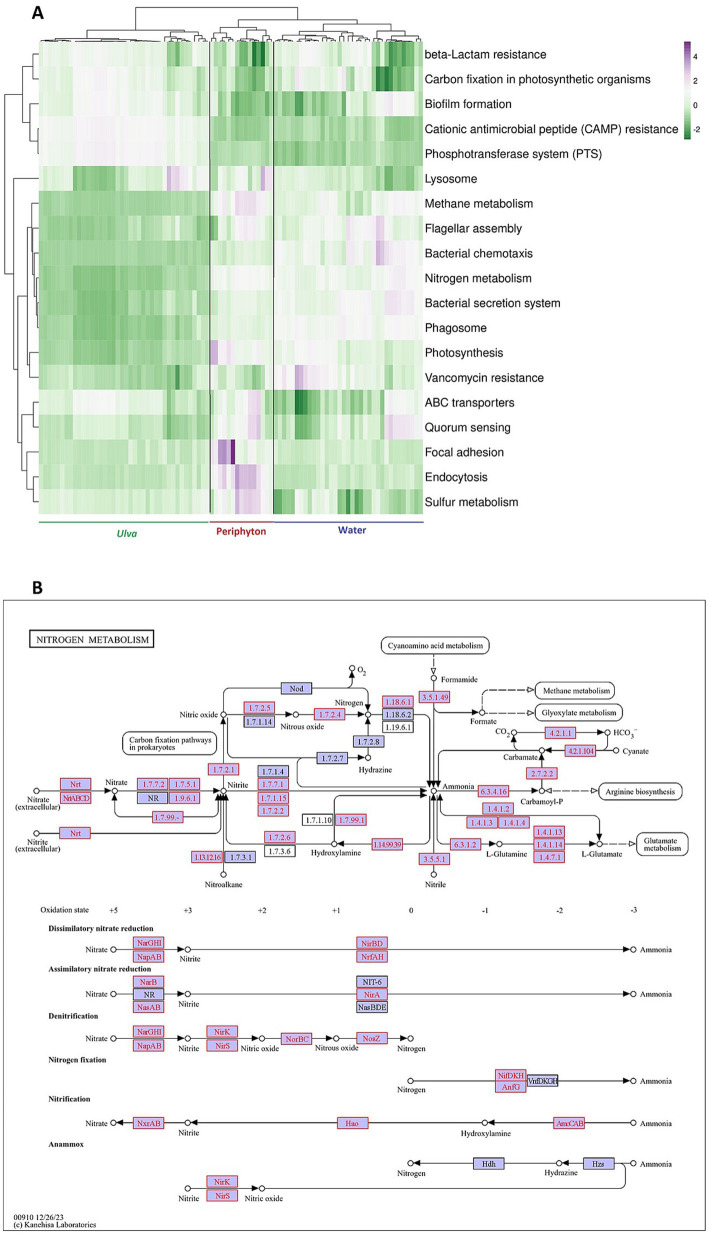
**(A)** a clustered heatmap showing some selected pathways regarding biofilm functions that were found in the microbial communities in Periphyton, *Ulva,* and water of the biofilter system. The colors range from green to purple, representing the lowest to highest KEGG orthologous gene abundance across all samples. **(B)** Predicted functional pathway of the nitrogen metabolism performed by microbial communities along different compartments in the biofilter. The map was created in the KEGG Orthology database (https://www.genome.jp/kegg/), where each box represents all the genes in the metabolism pathway. Boxes with red letters showed the genes identified in our current communities. Arrows indicate the direction of the metabolic process, ending with circles that represent targeted compounds.

Several critical KEGG pathways were observed for the microbial communities’ survival in marine periphyton, *Ulva*, and seawater, such as cell communication and motility, cellular community, energy metabolism, membrane transport, transport, and catabolism (Figure 7A). While several pathways, such as methane metabolism, flagellar assembly, bacterial chemotaxis, nitrogen metabolism, bacterial secretion system, phagosome, and photosynthesis, were more enriched in periphyton and water microbiome, they were more depleted in *Ulva*-associated microbial communities. Marine periphyton was primarily characterized by a high abundance of genes associated with ABC transporters, quorum sensing, focal adhesion, and endocytosis. Noticeably, regarding sulfur metabolism, periphyton obtained a significantly higher value of Shannon’s diversity index compared to *Ulva* and water microbiota (Supplementary Figure S5A). On the other hand, the *Ulva* microbiome was enriched in other pathways related to carbon fixation, biofilm formation, cationic antimicrobial peptide (CAMP) resistance, and Phosphotransferase system (PTS). Concerning nitrogen metabolism, a crucial process in the biofilter, the majority of genes involved in this process were present in our data, shown by boxes with red letters (Figure 7B). All the available modules for nitrogen metabolism were dissimilatory and assimilatory nitrate reduction (Nitrate to Ammonia), N fixation (Nitrogen to Ammonia), nitrification (Ammonia to Nitrate), and denitrification (nitrate to nitrogen) (Figure 7B). Especially, genes involved in nutrient removal (e.g., NapAB, NirS, and NosZ) were identified in the predicted functional pathway of the nitrogen metabolism performed by microbial communities along different compartments in the biofilter Figure 7B). Noticeably, significantly more KOs related to nitrogen metabolism in general and unique genes involved in different processes, such as dissimilatory nitrate reduction to ammonia (DNRA), denitrification, and nitrification, in particular, were found higher in periphyton (4.35%) and water microbiomes (4.3%) than in macroalgal microbiota (3.29%) (Supplementary Table S4, S5). Also, Shannon’s diversity index of nitrogen metabolism genes was higher in periphyton than in *Ulva* and water (Supplementary Figure S5B). All the unique genes in the nitrification process are not significantly different between all samples (Supplementary Table S6A). Yet, across all three habitats, DNRA obtained the highest relative abundance of unique genes, followed by denitrification and nitrification, respectively (Supplementary Table S6B). Overall, functional diversity (Shannon) was highest in *Ulva*. Still, genes associated with N and S metabolism were significantly enriched in the periphyton.

## 4 Discussion

Biofilters based on macroalgae and periphyton have been crucial parts of holistic mariculture systems, highlighting the continuous transfer of marine microorganisms to different compartments via the stream of fishpond effluent. This integrated biofilter offers a sustainable approach for enhancing mariculture wastewater quality by synergistically removing ammonia, nitrate, and phosphate (Guttman et al., 2018). However, the dynamics and functionality of the microbial community along the effluent treatment process over time are understudied. Knowing that environmental variables such as dissolved oxygen, temperature, nutrients gradients may influence the microbial diversity and metabolisms of the marine ecosystems at different scales (He et al., 2025; Yoon et al., 2025; Bick et al., 2025); in this study, we studied the microbial communities and predicted functions across the macroalgal *Ulva*-associated microbiota, periphyton, and the water microbiome over a five-week period, taking into account the ambient environment and nutrient flow.

Data from physical (pH, oxygen concentration, and temperature) and chemical measurements (TAN, NO3, PO4) in the biofilter culture tanks suggested that the local environment underlies microbial community patterns observed in *Ulva* and periphyton. Regarding community structure, each biofilter compartment harbored various bacterial groups that differed from those found in their ambient waters. Diversity-wise, the highest alpha diversity was observed in the periphyton microbial community, followed by the water microbiome and *Ulva*-associated microbiota. Marine periphyton supports abundant biofilm communities, where multiple species proliferate, including microorganisms that can reside and thrive on plastic substrates (phyla Proteobacteria, Bacteroidetes, and Cyanobacteria) (Sanli et al., 2015; Zhang et al., 2019). The result of prominent bacteria found in our biofilter is in agreement with findings about planktonic microorganisms in marine culture (Zhang et al., 2024). Macroalgae *Ulva fasciata*, on the other hand, collectively recruits its associated microbiome (including phyla Bacteroidetes, Planctomycetes, and Cyanobacteria) through a mutualistic relationship (Califano et al., 2020; Nguyen et al., 2023a), which is responsible for the growth and morphogenesis of *Ulva* (Weiss et al., 2017; Alsufyani et al., 2020; Wichard, 2023). As a medium, water allows a variety of species to inhabit (phyla Proteobacteria and Bacteroidetes) (Basili et al., 2020). Although the microbes that reside in water and substrates are distinct, the surrounding environment, in general, and water in particular, play a crucial role in microbial assembly within the biofilm community (Amaral-Zettler et al., 2020; Basili et al., 2020). Diversity-wise, due to the continuous water movement, its community cannot be as rich as the periphyton, but is higher than that of *Ulva*, where the selection force is very high. We also noticed a high prevalence of Order Vibrionales, which are well-known residents of seawaters (Imhoff, 2005), while their prevalence among *Ulva* and periphyton samples was insignificant. This could be explained by the inhibitory effect of periphyton and *Ulva* on *Vibrio* sp., suggesting that the biofilter contributes to overcoming pathogenic factors in the effluents and enhancing the well-being of cultured species (Lu et al., 2008; Kumar et al., 2017). Following the pattern of community compositional diversity, we also detected distinct functional diversity among the three habitats. However, the highest functional diversity was observed in *Ulva* microbiota, followed by periphyton and water microbiomes.

It is well known that environmental heterogeneity and hydraulic connectivity co-govern nitrogen metabolism by creating microhabitats that favour various microbial assembly processes (Chen et al., 2025). In particular, the two processes of denitrification and dissimilatory nitrate reduction to ammonium (DNRA) can coexist, with one process dominating the other depending on environmental gradients, such as dissolved oxygen and nitrate, across adjacent niches (Wang et al., 2024). In our biofilter system, many gene orthologs were identified in the data regarding the nutrient removal/ nitrogen metabolism pathway (e.g., NapAB, NirS, and NosZ), where gene abundance is significantly different between periphyton and *Ulva* compared to the culture waters. Especially, we found a higher prevalence of genes involved in denitrification in the periphyton microbial community than in *Ulva* and the water environments. This is noteworthy since denitrification prevailed in anaerobic submerged environments (such as biofilms) despite high oxygen concentration in the surrounding water (Eriksson, 2001). On the other hand, genes related to nitrification were not different among habitats, as ammonia oxidation widely occurs across the system. Notably, *Ulva* microbiota favors unique genes involved in DNRA, in which nitrate is converted to bioavailable ammonia, thereby enhancing macroalgal growth when nitrate uptake is restricted (Shahar et al., 2020). This evidence shows that environmental heterogeneity governs niche partitioning (DNRA versus denitrification) across the different compartments of the biofilter. Lastly, the targeted group of essential pathways related to biofilm formation and functioning (such as bacterial chemotaxis, bacterial secretion system, biofilm formation, and phosphotransferase system) showed significant separations among the three compartments in terms of relative gene abundance.

It is important to notice that the functional predictions generated by PICRUSt are based on 16S rRNA gene profiles and reference genomes. Thus, it provides only a prediction of community functional potential rather than direct measurements of genes or pathways. Given the specificity of marine environments, where many taxa lack closely related reference genomes and have unresolved taxonomy, the prediction accuracy could be lowered. What is more, our study did not aim to quantify microbial diffusion, as this requires specialized methodologies. However, we can assume that the rapid flow of water through the interconnected biofilters allows relatively high diffusion, which, in turn, is supported by significant differences in microbial assemblies and associated changes in environmental gradients (in our case, nutrient composition). While fully aware of the limitations of gene inference methods based on amplicon sequencing data as well as technical limitations such as diffusion quantification, the results presented in this research still supported the conclusion that each compartment was characterized by distinct bacterial communities and functions, despite the interconnection between *Ulva* and periphyton via water fluxes. All in all, our study aligns with other aquatic research (Yoon et al., 2025; Vitanza et al., 2025; He et al., 2025), highlighting the role of environmental heterogeneity in shaping microbial communities and their metabolism.

## 5 Conclusion

Environmental factors, in general, and nutrient flow, in particular, are known to drive the community assembly process. Our study on a unique biofilter system consisting of *Ulva fasciata* and marine periphyton, which are interconnected throughout continuous water fluxes, has provided an exceptional model to study the microbial dynamics in an engineered aquatic environment, a topic of theoretical and practical interest. We propose that environmental heterogeneity, as reflected in physicochemical parameters, has shaped the structure, dynamics, and function of microbial communities across various hosts and time periods. Predicted genes associated with the nitrogen and sulfur metabolism pathways were the highest in periphyton, followed by water and *Ulva*. Our findings contributed to a more comprehensive understanding of microbial ecology in a dynamic fluid system. It is suggested to quantify the diffusion of microorganisms and their impact on the community structure in future research, in order to draw more solid conclusions about microbial community assembly and dynamics in aquatic engineered environments.

## Data Availability

The data presented in this study are publicly available in the EMBL Nucleotide Sequence Database (ENA) repository under accession numbers PRJEB51318 (https://www.ebi.ac.uk/ena/browser/view/PRJEB51318) and PRJEB62134 (https://www.ebi.ac.uk/ena/browser/view/PRJEB62134).
