# Supplementary material for: Microbial dynamics along nutrient flow and removal in an integrated multitrophic aquaculture system
Source: Front Microbiol. 2026 Apr 21;17:1781931. doi: 10.3389/fmicb.2026.1781931 (PMC13139120; doi:10.3389/fmicb.2026.1781931)
Supplement: Supplementary file 1 [file Data_Sheet_1.docx]

Supplementary Material

**Microbial dynamics along nutrient flow and removal in an**

**integrated multitrophic aquaculture system**

Dzung Nguyen^1^, Ofer Ovadia^2,3^, Matan Masasa^4^, Andrea Tarnecki^5^, Nathan P. Brennan^6^, Nicole R. Rhody^6^, Kevan L. Main^6^, and Lior Guttman^7*^

# Supplementary Figures


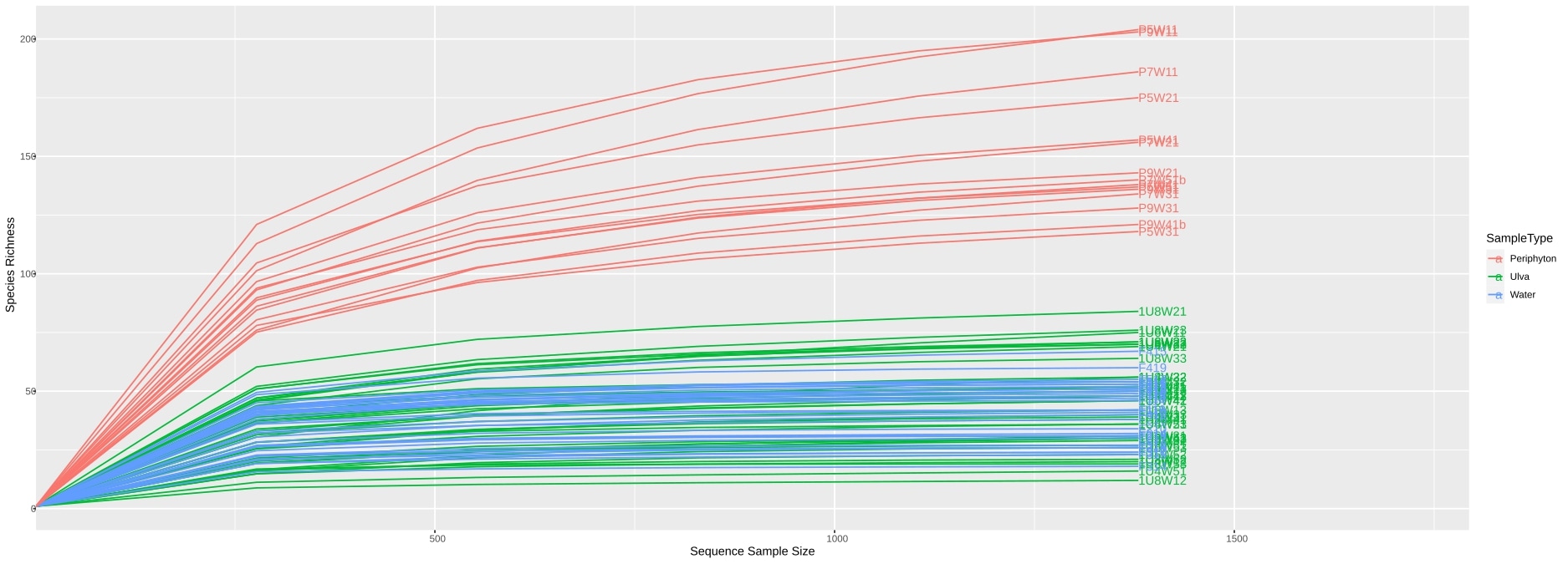


**Figure S1:** Rarefaction curves based on species richness of the microbial communities in *Ulva*, periphyton, and water of the biofilter. Samples were normalized to the minimum library size and colored based on different habitats.


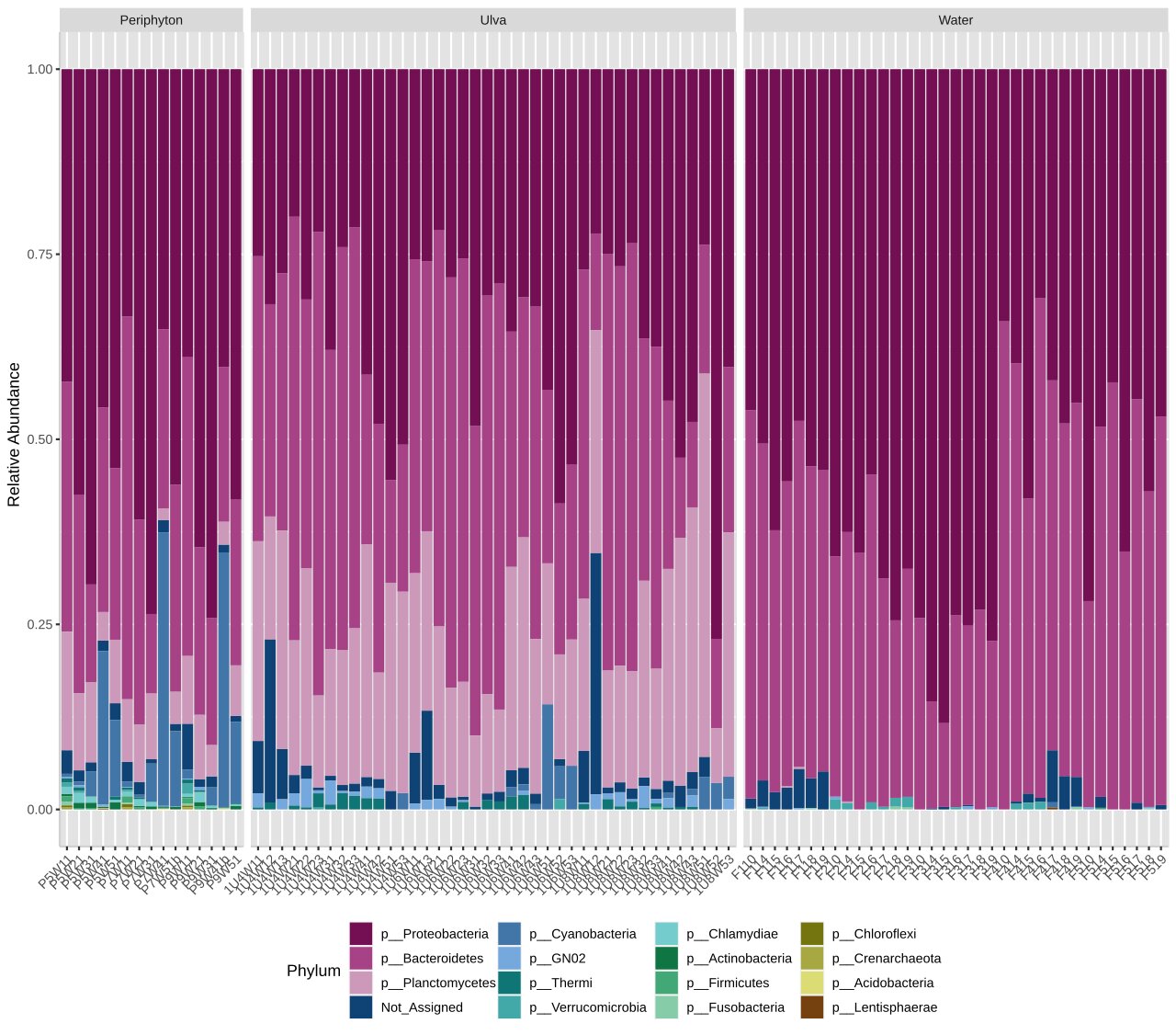

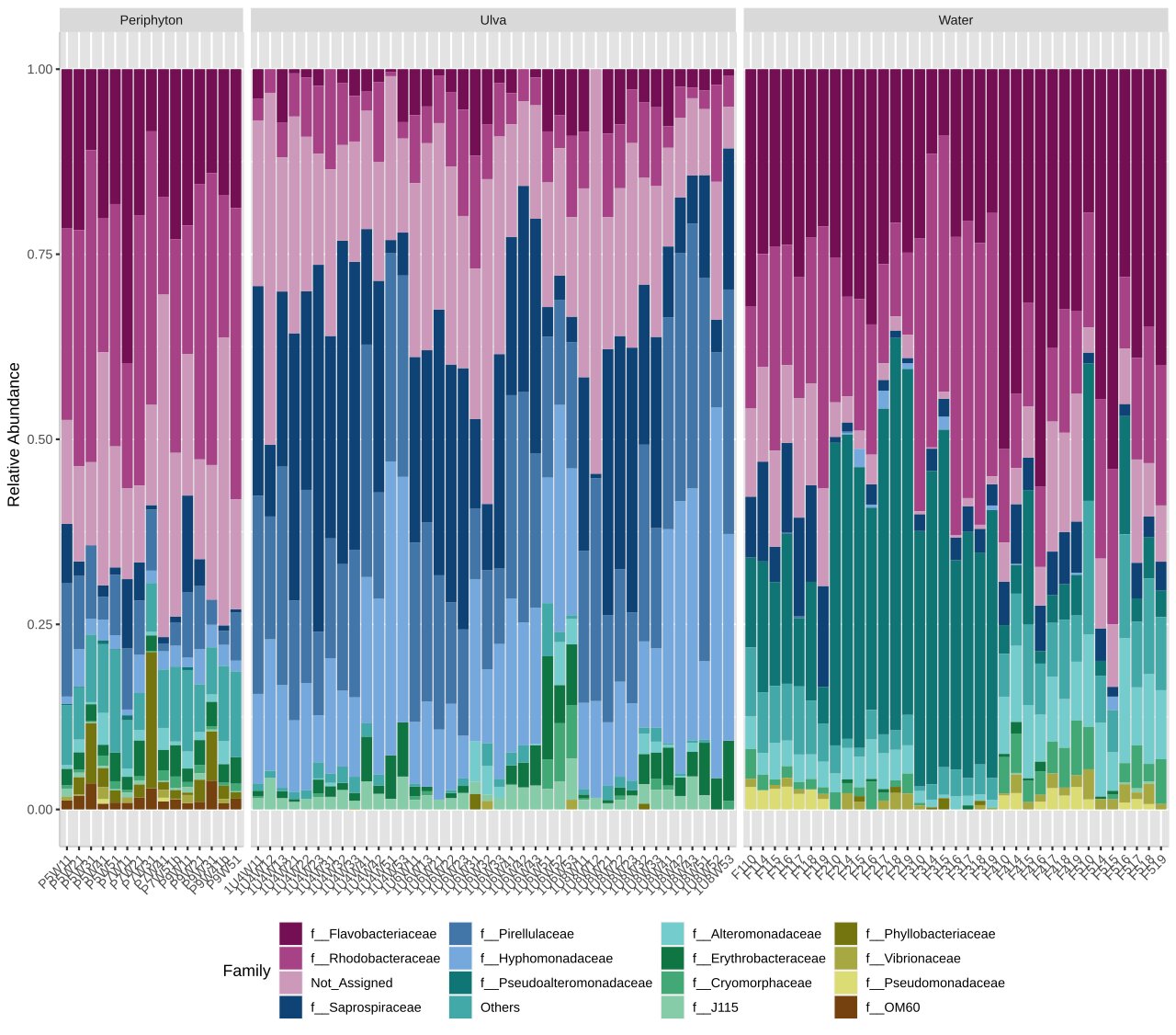


**Figure S2**: Relative abundance of the microbial community in the marine periphyton, *Ulva*, and water during five weeks of succession, shown by the relative abundance at phylum and family levels. Only the top 15 most abundant taxa are displayed at the family level.


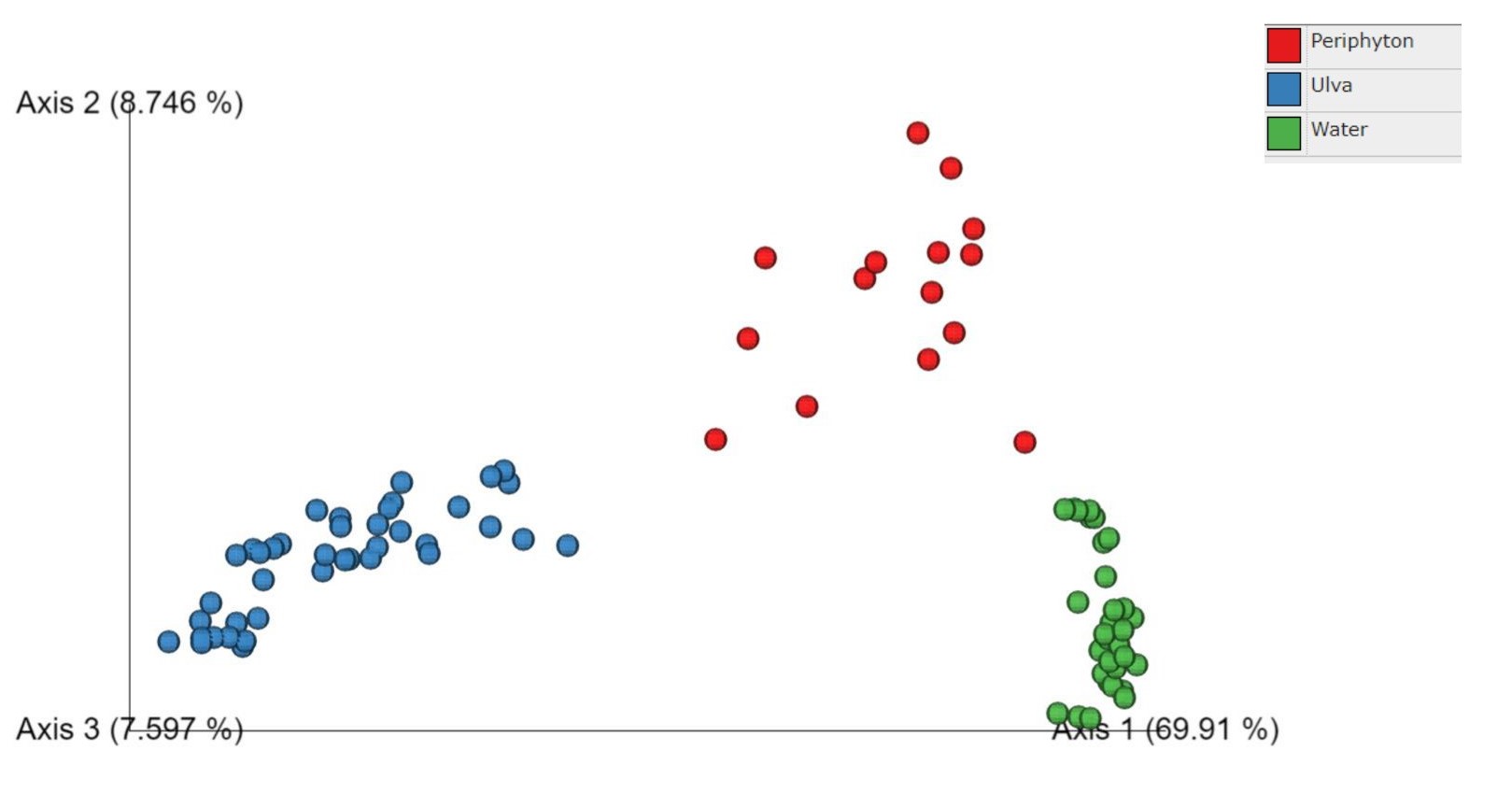
 **Figure S3:** Dissimilarities in microbial communities across three compartments (periphyton, *Ulva*, and water) based on UniFrac, which considers phylogenetic distance statistical test PERMANOVA resulted in p-value: 0.001.


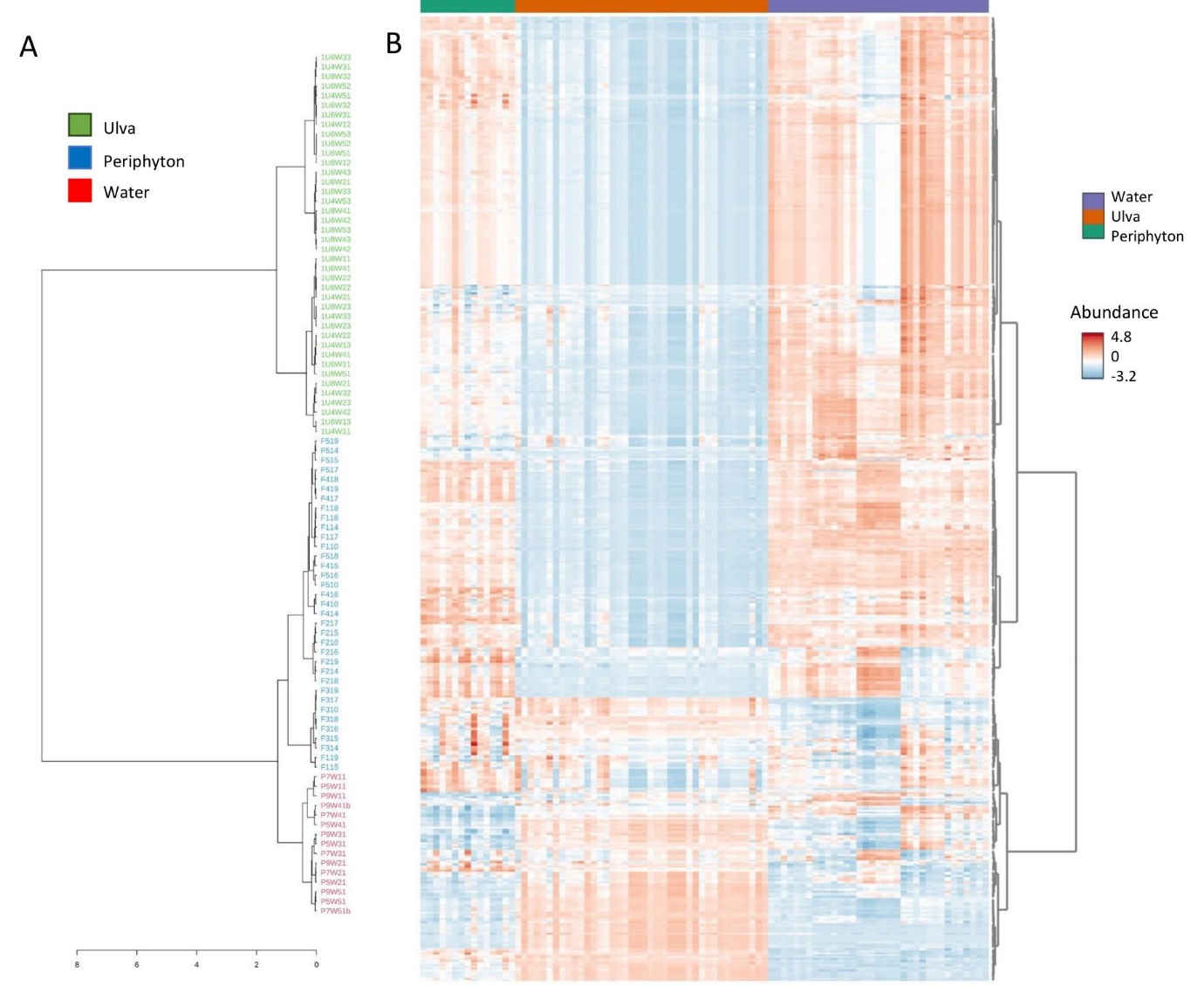


**Figure S4: A.** Dendrogram showing how samples belonging to different communities are clustered based on the Bray-Curtis index distance measure and Ward clustering algorithm. Other sources of samples are colored green (*Ulva* microbiota), blue (water), and red (periphyton). **B.** A clustered heatmap of the predicted functions found in the microbial community of periphyton, *Ulva* microbiota, and water, based on the KEGG Ortholog database. Colors denote each gene ortholog according to the number of KOs found in each sample, ranging from blue (low value) to orange (high value).

**A**
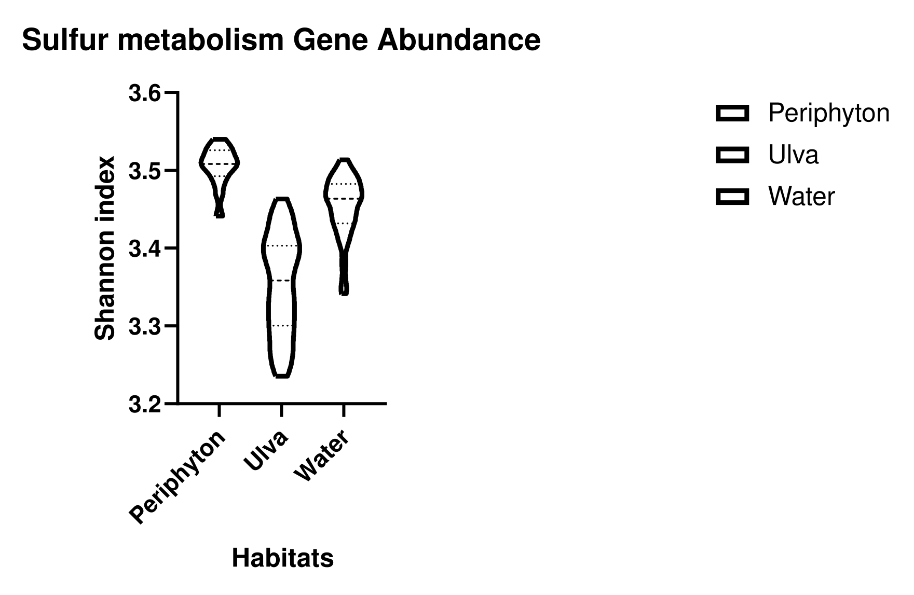
**B**
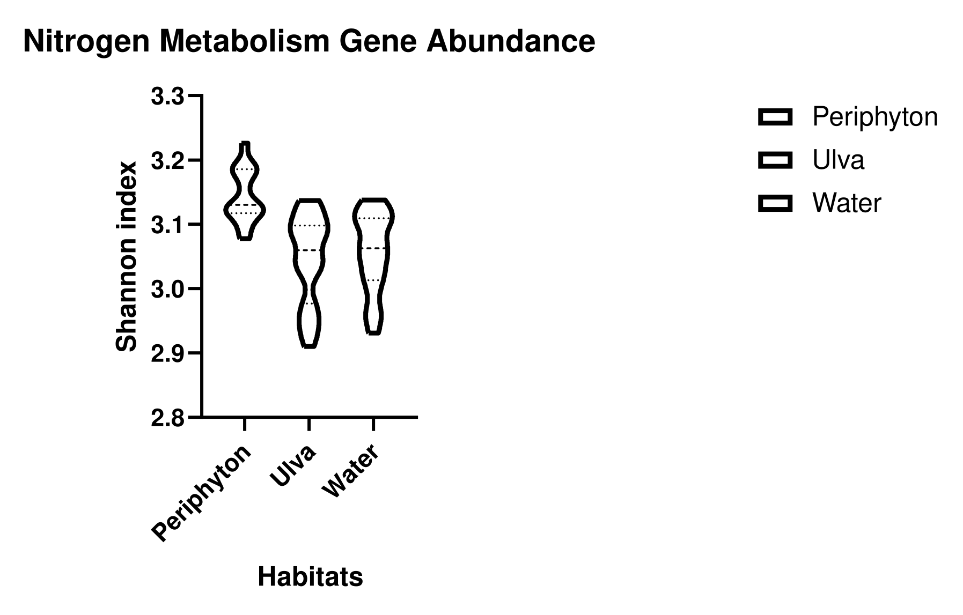


**Figure S5:** Alpha diversity based on Shannon’s Index was calculated for orthologous genes (relative abundance) involved in A. Sulfur and B. Nitrogen metabolisms. Dunnett’s multiple comparisons test shows significantly higher diversity in periphyton than in *Ulva* and water in both pathways (P<0.01).

# Supplementary Tables

**Table S1:** Physical (pH, oxygen concentration, and temperature) and chemical (TAN, NO3, PO4) measurements in the ambient water of the dual biofilter system consisting of upstream *Ulva fasciata* and marine periphyton. Physical measurements were done inside the cultivation tanks, while water samples for chemical analysis were taken from the water inlet and outlet of each tank.

| **Sample-ID** | **Patch type** | **Time** | **pH** | **O2 (%)** | **O2 (mg/l)** | **Temp (^o^C)** | **TAN/N (mg/l)** | **NO3/N (mg/l)** | **PO4/P (mg/l)** |
| --- | --- | --- | --- | --- | --- | --- | --- | --- | --- |
| P5W11 | Periphyton | Week1 | 7.97 | 107.7 | 7.52 | 21.3 | 0.57 | 0.64 | 0.2 |
| P5W21 | Periphyton | Week2 | 8.19 | 104.5 | 7.37 | 19.9 | 0.79 | 0.83 | 0.14 |
| P5W31 | Periphyton | Week3 | 8.39 | 136.2 | 9.86 | 18.8 | 1.36 | 1.92 | 0.26 |
| P5W41 | Periphyton | Week4 | 8.42 | 133.9 | 9.5 | 19.6 | 2.09 | 0.71 | 0.17 |
| P5W51 | Periphyton | Week5 | 8.29 | 138.3 | 9.78 | 20.3 | 1.55 | 2.16 | 0.03 |
| P7W11 | Periphyton | Week1 | 8.12 | 106.6 | 7.29 | 22 | 0.77 | 0.73 | 0.16 |
| P7W21 | Periphyton | Week2 | 8.23 | 121.8 | 8.72 | 20 | 0.8 | 0.92 | 0.1 |
| P7W31 | Periphyton | Week3 | 8.4 | 150.7 | 10.9 | 18.9 | 1.37 | 2.03 | 0.31 |
| P7W41 | Periphyton | Week4 | 8.4 | 131.2 | 9.29 | 19.8 | 1.83 | 0 | 0.07 |
| P7W51b | Periphyton | Week5 | 8.33 | 135.3 | 9.61 | 20.3 | 2.84 | 0.35 | 0.05 |
| P9W11 | Periphyton | Week1 | 8.26 | 111.6 | 7.68 | 21.6 | 0.68 | 0.65 | 0.16 |
| P9W21 | Periphyton | Week2 | 8.22 | 122.3 | 8.74 | 18.9 | 0.48 | 0.69 | 0.12 |
| P9W31 | Periphyton | Week3 | 8.39 | 139.6 | 10.1 | 18.8 | 1.35 | 2.04 | 0.39 |
| P9W41b | Periphyton | Week4 | 8.43 | 132.6 | 9.42 | 19.7 | 1.15 | 0.11 | 0.25 |
| P9W51 | Periphyton | Week5 | 8.34 | 134.7 | 9.56 | 20.1 | 1.56 | 0.3 | 0.13 |
| U4W1 | *Ulva* | Week1 | 7.96 | 122 | 8.37 | 22 | 1.69 | 1.57 | 0.33 |
| U4W2 | *Ulva* | Week2 | 7.94 | 105.5 | 7.37 | 20.8 | 0.9 | 0.52 | 0.12 |
| U4W3 | *Ulva* | Week3 | 8.08 | 108.9 | 7.76 | 19.8 | 1.82 | 1.93 | 0.31 |
| U4W4 | *Ulva* | Week4 | 7.97 | 119 | 8.37 | 20.2 | 2.38 | 0.23 | 0.13 |
| U4W5 | *Ulva* | Week5 | 8.04 | 118.7 | 8.32 | 20.8 | 2.08 | 1.87 | 0.19 |
| U6W1 | *Ulva* | Week1 | 7.97 | 113.4 | 7.75 | 22.1 | 1.64 | 1.52 | 0.29 |
| U6W2 | *Ulva* | Week2 | 7.99 | 99.8 | 6.91 | 20 | 0.92 | 0.56 | 0.12 |
| U6W3 | *Ulva* | Week3 | 8.07 | 106 | 7.58 | 19.5 | 1.81 | 1.93 | 0.24 |
| U6W4 | *Ulva* | Week4 | 8.01 | 110.1 | 7.74 | 20.2 | 1.65 | 0.18 | 0.13 |
| U6W5 | *Ulva* | Week5 | 8.01 | 117.9 | 8.28 | 20.6 | 1.3 | 1.26 | 0.09 |
| U8W1 | *Ulva* | Week1 | 7.98 | 128.4 | 8.76 | 22.1 | 1.64 | 1.53 | 0.31 |
| U8W2 | *Ulva* | Week2 | 7.97 | 105.5 | 7.67 | 18.8 | 0.85 | 0.54 | 0.14 |
| U8W3 | *Ulva* | Week3 | 8.08 | 108.5 | 7.76 | 19.4 | 1.8 | 1.99 | 0.24 |
| U8W4 | *Ulva* | Week4 | 7.95 | 119.5 | 8.41 | 20.1 | 1.87 | 0.21 | 0.35 |
| U8W5 | *Ulva* | Week5 | 8.02 | 122.4 | 8.6 | 20.5 | 1.44 | 0 | 0.05 |

**Table S2:** The relative abundance (%) of the microbial communities at the phyla level was found in three types of samples: periphyton, *Ulva*, and water, where the mean value was calculated for each taxon in each type of sample.

| **Phylum** | **Periphyton** | **Phylum** | ***Ulva*** | **Phylum** | **Water** |
| --- | --- | --- | --- | --- | --- |
| Proteobacteria | 53.61 | Bacteroidetes | 36.26 | Proteobacteria | 58.58 |
| Bacteroidetes | 26.03 | Proteobacteria | 35.27 | Bacteroidetes | 39.7 |
| Cyanobacteria | 9.18 | Planctomycetes | 22.24 | Verrucomicrobia | 0.27 |
| Planctomycetes | 7.5 | Cyanobacteria | 1.05 | GN02 | 0.05 |
| Chlamydiae | 0.52 | GN02 | 0.85 | Fusobacteria | 0.04 |
| Actinobacteria | 0.37 | Thermi | 0.59 | Cyanobacteria | 0.02 |
| Firmicutes | 0.23 | Verrucomicrobia | 0.04 | Planctomycetes | 0.02 |
| Thermi | 0.2 | Firmicutes | 0.01 | Lentisphaerae | 0.01 |
| Verrucomicrobia | 0.18 | Not_Assigned | 3.69 | Firmicutes | 0.01 |
| GN02 | 0.1 |  |  | Not_Assigned | 1.31 |
| Chloroflexi | 0.07 |  |  |  |  |
| Fusobacteria | 0.06 |  |  |  |  |
| Crenarchaeota | 0.05 |  |  |  |  |
| Acidobacteria | 0.03 |  |  |  |  |
| Lentisphaerae | 0.01 |  |  |  |  |
| Not_Assigned | 1.86 |  |  |  |  |

**Table S3:** The relative abundance (%) of all the bacterial orders found in three types of samples: periphyton, *Ulva*, and water. Each value was the mean value of samples collected for that sample type.

| **Order** | **Periphyton** | **Order** | ***Ulva*** | **Order** | **Water** |
| --- | --- | --- | --- | --- | --- |
| Rhodobacterales | 31.67 | Rhodobacterales | 26.87 | Flavobacteriales | 34.05 |
| Flavobacteriales | 20.30 | Pirellulales | 21.87 | Vibrionales | 21.77 |
| Pirellulales | 6.89 | Saprospirales | 21.23 | Rhodobacterales | 20.81 |
| Alteromonadales | 4.15 | Flavobacteriales | 4.90 | Alteromonadales | 6.49 |
| Rhizobiales | 3.57 | Alteromonadales | 3.50 | Saprospirales | 4.93 |
| Saprospirales | 3.18 | Sphingomonadales | 2.77 | Pseudomonadales | 1.66 |
| Sphingomonadales | 2.88 | Bacteroidales | 0.84 | Oceanospirillales | 1.37 |
| Chroococcales | 1.67 | Deinococcales | 0.59 | Rickettsiales | 1.01 |
| BD7_3 | 0.97 | Pseudanabaenales | 0.18 | Campylobacterales | 0.81 |
| Pseudanabaenales | 0.86 | Rhizobiales | 0.08 | Bacteroidales | 0.52 |
| Marinicellales | 0.82 | Vibrionales | 0.06 | Sphingomonadales | 0.18 |
| Cytophagales | 0.69 | Campylobacterales | 0.01 | Cytophagales | 0.17 |
| Vibrionales | 0.56 | Cytophagales | 0.01 | Rhizobiales | 0.11 |
| Rickettsiales | 0.36 | Chroococcales | 0.01 | Pirellulales | 0.02 |
| Bacteroidales | 0.22 | Rickettsiales | 0.01 | Not_Assigned | 3.73 |
| Deinococcales | 0.20 | Marinicellales | 0.00 | Others | 2.37 |
| Campylobacterales | 0.13 | Not_Assigned | 16.80 |  |  |
| Pseudomonadales | 0.08 | Others | 0.26 |  |  |
| Oceanospirillales | 0.04 |  |  |  |  |
| Not_Assigned | 16.08 |  |  |  |  |
| Others | 4.68 |  |  |  |  |

**Table S4:** Relative abundance (%) of KEGG pathways found across three types of habitats: periphyton, *Ulva*, and water under broader categories in bold letters.

| **Pathway** | **Periphyton** | ***Ulva*** | **Water** | **Total** |
| --- | --- | --- | --- | --- |
| **Cell Communication** |  |  |  |  |
| Focal adhesion | 0.012 | 0.001 | 0.002 | 0.014 |
| **Cell Motility** |  |  |  |  |
| Bacterial chemotaxis | 1.927 | 1.357 | 2.598 | 5.883 |
| Flagellar assembly | 6.004 | 5.064 | 6.244 | 17.312 |
| **Cellular community - prokaryotes** |  |  |  |  |
| Biofilm formation | 5.928 | 6.740 | 6.292 | 18.960 |
| Quorum sensing | 6.353 | 5.131 | 5.663 | 17.148 |
| **Drug resistance: antimicrobial** |  |  |  |  |
| beta-Lactam resistance | 1.484 | 1.630 | 1.580 | 4.694 |
| Cationic antimicrobial peptide (CAMP) resistance | 1.898 | 3.291 | 2.007 | 7.196 |
| Vancomycin resistance | 1.466 | 1.393 | 1.608 | 4.467 |
| **Energy Metabolism** |  |  |  |  |
| Carbon fixation in photosynthetic organisms | 4.483 | 4.682 | 4.568 | 13.733 |
| Methane metabolism | 5.811 | 4.618 | 5.347 | 15.777 |
| Nitrogen metabolism | 4.345 | 3.287 | 4.302 | 11.934 |
| Photosynthesis | 2.897 | 2.285 | 2.667 | 7.849 |
| Sulfur metabolism | 4.878 | 4.677 | 4.604 | 14.160 |
| **Membrane Transport** |  |  |  |  |
| ABC transporters | 24.222 | 23.406 | 21.305 | 68.933 |
| Bacterial secretion system | 6.017 | 4.673 | 6.871 | 17.561 |
| Phosphotransferase system (PTS) | 0.648 | 1.166 | 0.568 | 2.383 |
| **Transport and Catabolism** |  |  |  |  |
| Endocytosis | 0.038 | 0.002 | 0.004 | 0.044 |
| Lysosome | 1.033 | 0.859 | 0.684 | 2.576 |
| Phagosome | 2.094 | 1.483 | 2.456 | 6.034 |

**Table S5:** Multiple comparisons between different KEGG pathways found in microbial communities across three types of habitats, i.e., periphyton, *Ulva*, and water. A two-way ANOVA followed by Tukey’s post-hoc test was used for each pathway, with significant results highlighted in red.

| **Tukey's multiple comparisons test** | **Mean Diff.** | **95.00% CI of diff.** | **Signifi-cant?** | **Summary** | **Adjusted P-Value** |
| --- | --- | --- | --- | --- | --- |
| **ABC transporters** |  |  |  |  |  |
| Periphyton vs. *Ulva* | 0.1632 | -0.729 to 1.055 | No | ns | 0.8014 |
| Periphyton vs. Water | 0.5834 | 0.004 to 1.163 | Yes | * | 0.049 |
| *Ulva* vs. Water | 0.4202 | -0.405 to 1.246 | No | ns | 0.2763 |
| **Bacterial chemotaxis** |  |  |  |  |  |
| Periphyton vs. *Ulva* | 0.1141 | 0.060 to 0.168 | Yes | ** | 0.0037 |
| Periphyton vs. Water | -0.1342 | -0.231 to -0.0379 | Yes | * | 0.0167 |
| *Ulva* vs. Water | -0.2482 | -0.322 to -0.174 | Yes | *** | 0.0006 |
| **Bacterial secretion system** |  |  |  |  |  |
| Periphyton vs. *Ulva* | 0.2689 | 0.081 to 0.457 | Yes | * | 0.0154 |
| Periphyton vs. Water | -0.1708 | -0.316 to -0.025 | Yes | * | 0.03 |
| *Ulva* vs. Water | -0.4397 | -0.677 to -0.203 | Yes | ** | 0.006 |
| **beta-Lactam resistance** |  |  |  |  |  |
| Periphyton vs. *Ulva* | -0.0291 | -0.054 to -0.004 | Yes | * | 0.0293 |
| Periphyton vs. Water | -0.0192 | -0.045 to 0.007 | No | ns | 0.1212 |
| *Ulva* vs. Water | 0.0099 | -0.0278 to 0.048 | No | ns | 0.6499 |
| **Biofilm formation** |  |  |  |  |  |
| Periphyton vs. *Ulva* | -0.1623 | -0.278 to -0.046 | Yes | * | 0.0165 |
| Periphyton vs. Water | -0.0727 | -0.309 to 0.163 | No | ns | 0.5645 |
| *Ulva* vs. Water | 0.0896 | -0.061 to 0.240 | No | ns | 0.2007 |
| **Carbon fixation in photosynthetic organisms** |  |  |  |  |  |
| Periphyton vs. *Ulva* | -0.0398 | -0.072 to -0.007 | Yes | * | 0.0259 |
| Periphyton vs. Water | -0.0171 | -0.055 to 0.021 | No | ns | 0.3425 |
| *Ulva* vs. Water | 0.0227 | -0.037 to 0.0827 | No | ns | 0.4434 |
| **Cationic antimicrobial peptide (CAMP) resistance** |  |  |  |  |  |
| Periphyton vs. *Ulva* | -0.2787 | -0.342 to -0.215 | Yes | *** | 0.0002 |
| Periphyton vs. Water | -0.0217 | -0.071 to 0.0274 | No | ns | 0.3539 |
| *Ulva* vs. Water | 0.257 | 0.199 to 0.315 | Yes | *** | 0.0002 |
| **Endocytosis** |  |  |  |  |  |
| Periphyton vs. *Ulva* | 0.0072 | 0.002 to 0.012 | Yes | * | 0.0128 |
| Periphyton vs. Water | 0.0069 | 0.003 to 0.011 | Yes | ** | 0.0093 |
| *Ulva* vs. Water | -0.0002 | -0.001 to 0.001 | No | ns | 0.742 |
| **Flagellar assembly** |  |  |  |  |  |
| Periphyton vs. *Ulva* | 0.188 | -0.094 to 0.470 | No | ns | 0.1541 |
| Periphyton vs. Water | -0.0479 | -0.297 to 0.201 | No | ns | 0.7835 |
| *Ulva* vs. Water | -0.236 | -0.376 to -0.097 | Yes | ** | 0.0084 |
| **Focal adhesion** |  |  |  |  |  |
| Periphyton vs. *Ulva* | 0.0022 | -0.0004 to 0.005 | No | ns | 0.0836 |
| Periphyton vs. Water | 0.0020 | -0.0007 to 0.005 | No | ns | 0.1205 |
| *Ulva* vs. Water | -0.0002 | -0.0005 to 0.0002 | No | ns | 0.3084 |
| **Lysosome** |  |  |  |  |  |
| Periphyton vs. *Ulva* | 0.0347 | -0.052 to 0.122 | No | ns | 0.4139 |
| Periphyton vs. Water | 0.0699 | -0.060 to 0.199 | No | ns | 0.2461 |
| *Ulva* vs. Water | 0.0352 | -0.020 to 0.090 | No | ns | 0.1685 |
| **Methane metabolism** |  |  |  |  |  |
| Periphyton vs. *Ulva* | 0.2385 | 0.111 to 0.366 | Yes | ** | 0.0058 |
| Periphyton vs. Water | 0.0927 | -0.068 to 0.254 | No | ns | 0.2158 |
| *Ulva* vs. Water | -0.1458 | -0.259 to -0.032 | Yes | * | 0.0221 |
| **Nitrogen metabolism** |  |  |  |  |  |
| Periphyton vs. *Ulva* | 0.2116 | 0.169 to 0.254 | Yes | *** | 0.0001 |
| Periphyton vs. Water | 0.0085 | -0.066 to 0.083 | No | ns | 0.9164 |
| *Ulva* vs. Water | -0.2031 | -0.287 to -0.120 | Yes | ** | 0.0022 |
| **Phagosome** |  |  |  |  |  |
| Periphyton vs. *Ulva* | 0.1223 | 0.0792 to 0.165 | Yes | ** | 0.0012 |
| Periphyton vs. Water | -0.0724 | -0.116 to -0.030 | Yes | ** | 0.0086 |
| *Ulva* vs. Water | -0.1947 | -0.217 to -0.172 | Yes | **** | <0.0001 |
| **Phosphotransferase system (PTS)** |  |  |  |  |  |
| Periphyton vs. *Ulva* | -0.1036 | -0.135 to -0.073 | Yes | *** | 0.0006 |
| Periphyton vs. Water | 0.0161 | 0.003 to 0.029 | Yes | * | 0.0237 |
| *Ulva* vs. Water | 0.1197 | 0.091 to 0.148 | Yes | *** | 0.0003 |
| **Photosynthesis** |  |  |  |  |  |
| Periphyton vs. *Ulva* | 0.1224 | 0.040 to 0.205 | Yes | * | 0.0133 |
| Periphyton vs. Water | 0.0459 | -0.011 to 0.103 | No | ns | 0.0928 |
| *Ulva* vs. Water | -0.0765 | -0.120 to -0.033 | Yes | ** | 0.0074 |
| **Quorum sensing** |  |  |  |  |  |
| Periphyton vs. *Ulva* | 0.2445 | -0.043 to 0.532 | No | ns | 0.0808 |
| Periphyton vs. Water | 0.1381 | -0.066 to 0.342 | No | ns | 0.1492 |
| *Ulva* vs. Water | -0.1064 | -0.431 to 0.218 | No | ns | 0.5292 |
| **Sulfur metabolism** |  |  |  |  |  |
| Periphyton vs. *Ulva* | 0.0402 | -0.0004 to 0.081 | No | ns | 0.0518 |
| Periphyton vs. Water | 0.0549 | -0.016 to 0.126 | No | ns | 0.1071 |
| *Ulva* vs. Water | 0.0146 | -0.023 to 0.053 | No | ns | 0.4341 |
| **Vancomycin resistance** |  |  |  |  |  |
| Periphyton vs. *Ulva* | 0.0147 | -0.020 to 0.050 | No | ns | 0.3774 |
| Periphyton vs. Water | -0.0284 | -0.043 to -0.014 | Yes | ** | 0.0049 |
| *Ulva* vs. Water | -0.0432 | -0.090 to 0.004 | No | ns | 0.0667 |

**Table S6A:** Tukey’s posthoc test results show how orthologous genes related to Nitrogen metabolism pathways, such as dissimilatory nitrate reduction, nitrification, and denitrification, differentiate between three types of samples (periphyton, *Ulva*, and water microbiomes). The table was arranged to present the unique genes of these three pathways first. Any genes belonging to more than one pathway are presented in the “Others” section. Red color denotes highlights for analysis (read in the text of the main manuscript).

| **Metabolism Pathway** | **Related KEGG Orthology** | **Mean Diff.** | **95.00% CI of diff.** | **Significant?** | **Summary** | **Adjusted P Value** |
| --- | --- | --- | --- | --- | --- | --- |
| **Dissimilatory nitrate reduction to ammonia (DNRA)** | **K00362_Dissimilatory nitrate reduction** |  |  |  |  |  |
|  | Periphyton vs. Ulva | 0.0029 | -0.002052 to 0.007902 | No | ns | 0.2062 |
|  | Periphyton vs. Water | 0.0001 | -0.006179 to 0.006401 | No | ns | 0.9978 |
|  | Ulva vs. Water | -0.0028 | -0.004751 to -0.0008773 | Yes | * | 0.0144 |
|  | **K00363_Dissimilatory nitrate reduction** |  |  |  |  |  |
|  | Periphyton vs. Ulva | 0.0022 | -0.004218 to 0.008610 | No | ns | 0.5037 |
|  | Periphyton vs. Water | 0.0039 | -0.003542 to 0.01144 | No | ns | 0.2583 |
|  | Ulva vs. Water | 0.0018 | 0.0002634 to 0.003243 | Yes | * | 0.0297 |
|  | **K15876_Dissimilatory nitrate reduction** |  |  |  |  |  |
|  | Periphyton vs. Ulva | 0.0009 | -0.0006938 to 0.002420 | No | ns | 0.2334 |
|  | Periphyton vs. Water | 0.0007 | -0.001090 to 0.002420 | No | ns | 0.444 |
|  | Ulva vs. Water | -0.0002 | -0.0006889 to 0.0002925 | No | ns | 0.4063 |
|  | **K03385_Dissimilatory nitrate reduction** |  |  |  |  |  |
|  | Periphyton vs. Ulva | 0.0010 | -0.0007108 to 0.002632 | No | ns | 0.2163 |
|  | Periphyton vs. Water | 0.0006 | -0.001332 to 0.002467 | No | ns | 0.5813 |
|  | Ulva vs. Water | -0.0004 | -0.001023 to 0.0002362 | No | ns | 0.1798 |
| **Denitrification (nitrate => nitrogen)** | **K00368_Denitrification** |  |  |  |  |  |
|  | Periphyton vs. Ulva | 0.0011 | 1.910e-005 to 0.002086 | Yes | * | 0.0472 |
|  | Periphyton vs. Water | 0.0004 | -0.001022 to 0.001803 | No | ns | 0.6234 |
|  | Ulva vs. Water | -0.0007 | -0.001083 to -0.0002423 | Yes | * | 0.0108 |
|  | **K00376_Denitrification** |  |  |  |  |  |
|  | Periphyton vs. Ulva | 0.0039 | -0.0003608 to 0.008180 | No | ns | 0.0654 |
|  | Periphyton vs. Water | 0.0023 | -0.001580 to 0.006187 | No | ns | 0.202 |
|  | Ulva vs. Water | -0.0016 | -0.002048 to -0.001164 | Yes | *** | 0.0005 |
|  | **K02305_Denitrification** |  |  |  |  |  |
|  | Periphyton vs. Ulva | 0.0029 | -0.0002823 to 0.006046 | No | ns | 0.0664 |
|  | Periphyton vs. Water | 0.0018 | -0.001033 to 0.004602 | No | ns | 0.1742 |
|  | Ulva vs. Water | -0.0011 | -0.001533 to -0.0006609 | Yes | ** | 0.0019 |
|  | **K04561_Denitrification** |  |  |  |  |  |
|  | Periphyton vs. Ulva | 0.0031 | -0.0003458 to 0.006614 | No | ns | 0.0686 |
|  | Periphyton vs. Water | 0.0016 | -0.001838 to 0.004996 | No | ns | 0.3287 |
|  | Ulva vs. Water | -0.0016 | -0.001925 to -0.001186 | Yes | *** | 0.0003 |
|  | **K15864_Denitrification** |  |  |  |  |  |
|  | Periphyton vs. Ulva | 0.0011 | -0.0001073 to 0.002332 | No | ns | 0.0661 |
|  | Periphyton vs. Water | 0.0008 | -0.0005569 to 0.002132 | No | ns | 0.2076 |
|  | Ulva vs. Water | -0.0003 | -0.0005410 to -0.0001078 | Yes | * | 0.013 |
| **Nitrification (ammonia => nitrite)** | **K10946 _Nitrification** |  |  |  |  |  |
|  | Periphyton vs. Ulva | 0.0003 | -6.463e-005 to 0.0005760 | No | ns | 0.0968 |
|  | Periphyton vs. Water | 0.0003 | -6.352e-005 to 0.0005675 | No | ns | 0.0966 |
|  | Ulva vs. Water | 0.0000 | -1.075e-005 to 3.372e-006 | No | ns | 0.2629 |
|  | **K10535_Nitrification** |  |  |  |  |  |
|  | Periphyton vs. Ulva | 0.0001 | -5.274e-005 to 0.0003417 | No | ns | 0.1218 |
|  | Periphyton vs. Water | 0.0001 | -5.313e-005 to 0.0003394 | No | ns | 0.1232 |
|  | Ulva vs. Water | 0.0000 | -5.573e-006 to 2.853e-006 | No | ns | 0.5378 |
|  | **K10944_Nitrification** |  |  |  |  |  |
|  | Periphyton vs. Ulva | 0.0001 | -2.850e-005 to 0.0002130 | No | ns | 0.109 |
|  | Periphyton vs. Water | 0.0001 | -2.755e-005 to 0.0002056 | No | ns | 0.1091 |
|  | Ulva vs. Water | 0.0000 | -8.934e-006 to 2.520e-006 | No | ns | 0.2286 |
|  | **K10945_Nitrification** |  |  |  |  |  |
|  | Periphyton vs. Ulva | 0.0001 | -2.845e-005 to 0.0002130 | No | ns | 0.1089 |
|  | Periphyton vs. Water | 0.0001 | -2.768e-005 to 0.0002057 | No | ns | 0.1095 |
|  | Ulva vs. Water | 0.0000 | -8.965e-006 to 2.482e-006 | No | ns | 0.2233 |
| **Others** | **K02567_Denitrification & Dissimilatory nitrate reduction** |  |  |  |  |  |
|  | Periphyton vs. Ulva | 0.0012 | -0.0003702 to 0.002776 | No | ns | 0.1088 |
|  | Periphyton vs. Water | 0.0003 | -0.001578 to 0.002115 | No | ns | 0.8669 |
|  | Ulva vs. Water | -0.0009 | -0.001632 to -0.0002365 | Yes | * | 0.0192 |
|  | **K00370_Denitrification & Dissimilatory nitrate reduction** |  |  |  |  |  |
|  | Periphyton vs. Ulva | 0.0011 | -0.002033 to 0.004184 | No | ns | 0.4977 |
|  | Periphyton vs. Water | 0.0020 | -0.0007852 to 0.004773 | No | ns | 0.1285 |
|  | Ulva vs. Water | 0.0009 | -0.0001948 to 0.002032 | No | ns | 0.0883 |
|  | **K00371_Denitrification & Dissimilatory nitrate reduction** |  |  |  |  |  |
|  | Periphyton vs. Ulva | 0.0010 | -0.002054 to 0.004117 | No | ns | 0.5175 |
|  | Periphyton vs. Water | 0.0018 | -0.0008412 to 0.004474 | No | ns | 0.1452 |
|  | Ulva vs. Water | 0.0008 | -0.0002185 to 0.001788 | No | ns | 0.1023 |
|  | **K00374_Denitrification & Dissimilatory nitrate reduction** |  |  |  |  |  |
|  | Periphyton vs. Ulva | 0.0010 | -0.002040 to 0.004076 | No | ns | 0.5199 |
|  | Periphyton vs. Water | 0.0019 | -0.0008469 to 0.004631 | No | ns | 0.1414 |
|  | Ulva vs. Water | 0.0009 | -0.0002349 to 0.001982 | No | ns | 0.1002 |
|  | **K02567_Denitrification & Dissimilatory nitrate reduction** |  |  |  |  |  |
|  | Periphyton vs. Ulva | 0.0012 | -0.0003702 to 0.002776 | No | ns | 0.1088 |
|  | Periphyton vs. Water | 0.0003 | -0.001578 to 0.002115 | No | ns | 0.8669 |
|  | Ulva vs. Water | -0.0009 | -0.001632 to -0.0002365 | Yes | * | 0.0192 |
|  | **K02568_Denitrification & Dissimilatory nitrate reduction** |  |  |  |  |  |
|  | Periphyton vs. Ulva | 0.0011 | -0.0003800 to 0.002577 | No | ns | 0.1174 |
|  | Periphyton vs. Water | 0.0002 | -0.001595 to 0.001913 | No | ns | 0.9448 |
|  | Ulva vs. Water | -0.0009 | -0.001634 to -0.0002446 | Yes | * | 0.0186 |

**Table 6B:** Relative abundance of total genes exclusively existed in one of the habitats, under different processes (dissimilatory nitrate reduction, denitrification, and nitrification)

|  | **Peri** | **Ulva** | **Water** |
| --- | --- | --- | --- |
| **Dissimilatory nitrate reduction to ammonia - DNRA** | 0.00104 | 0.000467 | 0.000548 |
| **Denitrification**  **(nitrate to nitrogen)** | 0.000672 | 3.1E-05 | 0.00024 |
| **Nitrification** | 2.39E-05 | 1.56E-07 | 7.47E-07 |
